# Genome-Wide Association Analysis in Asthma Subjects Identifies *SPATS2L* as a Novel Bronchodilator Response Gene

**DOI:** 10.1371/journal.pgen.1002824

**Published:** 2012-07-05

**Authors:** Blanca E. Himes, Xiaofeng Jiang, Ruoxi Hu, Ann C. Wu, Jessica A. Lasky-Su, Barbara J. Klanderman, John Ziniti, Jody Senter-Sylvia, John J. Lima, Charles G. Irvin, Stephen P. Peters, Deborah A. Meyers, Eugene R. Bleecker, Michiaki Kubo, Mayumi Tamari, Yusuke Nakamura, Stanley J. Szefler, Robert F. Lemanske, Robert S. Zeiger, Robert C. Strunk, Fernando D. Martinez, John P. Hanrahan, Gerard H. Koppelman, Dirkje S. Postma, Maartje A. E. Nieuwenhuis, Judith M. Vonk, Reynold A. Panettieri, Amy Markezich, Elliot Israel, Vincent J. Carey, Kelan G. Tantisira, Augusto A. Litonjua, Quan Lu, Scott T. Weiss

**Affiliations:** 1 Channing Laboratory, Brigham and Women's Hospital and Harvard Medical School, Boston, Massachusetts, United States of America; 2 Children's Hospital Informatics Program, Boston, Massachusetts, United States of America; 3 Partners HealthCare Center for Personalized Genetic Medicine, Harvard Medical School, Boston, Massachusetts, United States of America; 4 Program in Molecular and Integrative Physiological Sciences, Department of Environmental Health, Harvard School of Public Health, Boston, Massachusetts, United States of America; 5 Department of Population Medicine, Harvard Pilgrim Health Care and Harvard Medical School, Boston, Massachusetts, United States of America; 6 Nemours Children's Clinic, Center for Pharmacogenomics and Translational Research, Jacksonville, Florida, United States of America; 7 Vermont Lung Center, Department of Medicine and Physiology, University of Vermont, Burlington, Vermont, United States of America; 8 Center for Genomics and Personalized Medicine Research, Wake Forest School of Medicine, Winston-Salem, North Carolina, United States of America; 9 RIKEN Center for Genomic Medicine, Kanagawa, Japan; 10 Laboratory of Molecular Medicine, The Institute of Medical Science, The University of Tokyo, Tokyo, Japan; 11 National Jewish Health and University of Colorado Denver School of Medicine, Denver, Colorado, United States of America; 12 Clinical Science Center, University of Wisconsin, Madison, Wisconsin, United States of America; 13 Kaiser Permanente Southern California Region, San Diego, California, United States of America; 14 Washington University School of Medicine, St. Louis, Missouri, United States of America; 15 Arizona Respiratory Center, University of Arizona, College of Medicine, Tucson, Arizona, United States of America; 16 Pulmatrix, Lexington, Massachusetts, United States of America; 17 Department of Pediatric Pulmonology and Pediatric Allergology, Beatrix Children's Hospital, GRIAC Research Institute, University of Groningen, University Medical Center Groningen, The Netherlands; 18 Department of Pulmonology and Tuberculosis, GRIAC Research Institute, University of Groningen, University Medical Center Groningen, The Netherlands; 19 Department of Epidemiology, GRIAC Research Institute, University of Groningen, University Medical Center Groningen, The Netherlands; 20 Pulmonary, Allergy, and Critical Care Division, University of Pennsylvania, Philadelphia, Pennsylvania, United States of America; 21 Overlake Internal Medicine Associates, Bellevue, Washington, United States of America; 22 Pulmonary and Critical Care Division, Brigham and Women's Hospital and Harvard Medical School, Boston, Massachusetts, United States of America; University of Chicago, United States of America

## Abstract

Bronchodilator response (BDR) is an important asthma phenotype that measures reversibility of airway obstruction by comparing lung function (i.e. FEV_1_) before and after the administration of a short-acting β_2_-agonist, the most common rescue medications used for the treatment of asthma. BDR also serves as a test of β_2_-agonist efficacy. BDR is a complex trait that is partly under genetic control. A genome-wide association study (GWAS) of BDR, quantified as percent change in baseline FEV_1_ after administration of a β_2_-agonist, was performed with 1,644 non-Hispanic white asthmatic subjects from six drug clinical trials: CAMP, LOCCS, LODO, a medication trial conducted by Sepracor, CARE, and ACRN. Data for 469,884 single-nucleotide polymorphisms (SNPs) were used to measure the association of SNPs with BDR using a linear regression model, while adjusting for age, sex, and height. Replication of primary P-values was attempted in 501 white subjects from SARP and 550 white subjects from DAG. Experimental evidence supporting the top gene was obtained via siRNA knockdown and Western blotting analyses. The lowest overall combined P-value was 9.7E-07 for SNP rs295137, near the *SPATS2L* gene. Among subjects in the primary analysis, those with rs295137 TT genotype had a median BDR of 16.0 (IQR = [6.2, 32.4]), while those with CC or TC genotypes had a median BDR of 10.9 (IQR = [5.0, 22.2]). *SPATS2L* mRNA knockdown resulted in increased β_2_-adrenergic receptor levels. Our results suggest that *SPATS2L* may be an important regulator of β_2_-adrenergic receptor down-regulation and that there is promise in gaining a better understanding of the biological mechanisms of differential response to β_2_-agonists through GWAS.

## Author Summary

Bronchodilator response (BDR) is an important asthma phenotype that measures reversibility of airway obstruction by comparing lung function before and after the administration of short-acting β_2_-agonists, common medications used for asthma treatment. We performed a genome-wide association study of BDR with 1,644 white asthmatic subjects from six drug clinical trials and attempted to replicate these findings in 1,051 white subjects from two independent cohorts. The most significant associated variant was near the *SPATS2L* gene. We knocked down *SPATS2L* mRNA in human airway smooth muscle cells and found that β_2_-adrenergic receptor levels increased, suggesting that *SPATS2L* may be a regulator of BDR. Our results highlight the promise of pursuing GWAS results that do not necessarily reach genome-wide significance and are an example of how results from pharmacogenetic GWAS can be studied functionally.

## Introduction

Asthma is a chronic respiratory disease that affects over 20 million Americans and 300 million people worldwide [1], [2]. A hallmark characteristic of asthma is reversible airway obstruction, which is commonly measured via a bronchodilator response (BDR) test, in which the reduction of bronchoconstriction after administration of a short-acting reliever drug is quantified [3]. β_2_-agonists, the most common short-acting reliever drugs used during BDR tests and for asthma therapy, act in part by stimulating β_2_-adrenergic receptors (β_2_ARs) on airway smooth muscle cells to reduce bronchoconstriction via subsequent increases in cyclic adenosine monophosphate (cAMP) and protein kinase A (PKA) [3]. Although a comprehensive pathophysiologic understanding of BDR has not been obtained, it is a complex trait involving interactions among various tissues and cells, including inflammatory [4], airway epithelium [5], smooth muscle [6], and the autonomic nervous system [7]. In addition to being used for the diagnosis of asthma, BDR tests can be used to measure whether inhaled β_2_-agonists are effective in patients. Although short-acting β_2_-agonists are widely used clinically as asthma rescue medications, they are variably efficacious among patients [8]. Studying BDR may thus provide information regarding both the pathophysiology and pharmacogenetics of asthma.

The search for genetic variants that modify asthma susceptibility has resulted in the most recent multi-center asthma genome-wide association studies (GWAS) providing strong statistical evidence for the association of many genes, including the *IKZF3-ZPBP2-GSDMB-ORMDL3* locus, *HLA-DQ*, *IL1RL1*, *IL18RL1*, *IL33*, *TSLP*, *SLC22A5*, *SMAD3*, and *RORA*, with asthma [9], [10]. Functional experiments to identify the role that these genes play in asthma pathophysiology are hindered by the complexity of the asthma phenotype. Familial aggregation [11] and genetic association studies [12] have provided suggestive evidence for a genetic contribution to interindividual differences in BDR. Candidate genes reported to be associated with BDR include β_2_-adrenergic receptor (*ADRB2*) [13], [14], adenylyl cyclase type 9 (*ADCY9*) [15], corticotrophin-releasing hormone receptor 2 (*CRHR2*) [16], and arginase 1 (*ARG1*) [17], [18]. While BDR is a complex phenotype, functional studies of BDR candidate genes are simpler than those for a general asthma phenotype because this pharmacogenetic phenotype can be readily simulated *in vitro* via stimulation of cells with β_2_-agonists.

In this study, we performed a GWAS of BDR in 1,644 non-Hispanic white asthmatics and found that the strongest evidence of association with BDR was at variants near the Homo sapiens spermatogenesis associated, serine-rich 2-like (*SPATS2L*) gene. We attempted to replicate the primary findings in two independent populations and investigated the function of *SPATS2L* via mRNA knockdown experiments and found evidence to support its involvement in BDR.

## Results

### Primary BDR GWAS

**Figure 1:**
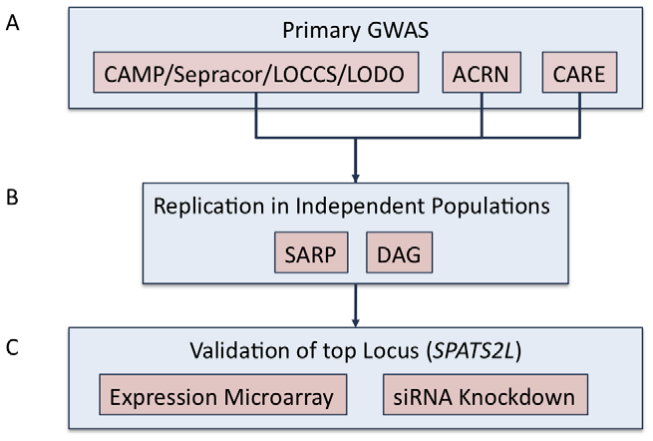
Study overview. (A) Primary GWAS was conducted using subjects from CAMP, LOCCS, LODO, Sepracor, ACRN, and CARE cohort. Samples genotyped on Illumina platforms (i.e. CAMP/LOCCS/LODO/Sepracor) were pooled and analyzed first. Results from samples genotyped on Affymetrix platforms were analyzed separately and then combined to obtain the primary GWAS results. 1000GP imputed data was utilized to expand the primary GWAS association results. (B) Replication of the top (i.e. P-value<1E-04) SNPs from the primary GWAS were attempted in two independent populations: SARP and DAG. (C) The *SPATS2L* gene was selected for functional validation based on nominal replication of association results in SARP and analysis of publicly available resources.

Figure 1 is an overview of our study design. Characteristics of the subjects used in the primary GWAS are provided in Table 1. We utilized 1,644 non-Hispanic white subjects from six clinical trials to measure the association of SNPs to BDR. After QC filters, 469,884 SNPs genotyped in CAMP/LOCCS/LODO/Sepracor and either genotyped or imputed in CARE and ACRN were used to test for the association of SNPs to BDR. We utilized genotyped SNPs for CAMP/LOCCS/LODO/Sepracor because these cohorts, who were all genotyped using Illumina platforms, had the largest sample size. Due to the poor overlap of Illumina and Affymetrix platform SNPs, we utilized HapMap Phase 2 imputed SNPs for CARE and ACRN, so that the maximal number of SNPs in all cohorts could be analyzed. The quantile-quantile (QQ) and Manhattan plots revealed that the distribution of association P-values was similar to that expected for a null distribution and that no P-values met genome-wide statistically significant levels (Figures S1 and S2). To expand the primary association results further, all SNPs available in the June 2010 release of the 1000 Genome Project (1000GP) data were imputed using MaCH in each of the three primary groups of genotype data and overall BDR GWAS results were re-computed. Among SNPs contained in the primary GWAS, imputed and genotyped P-values were similar, particularly for those with low P-values (Figure S3). Some imputed regions had P-values lower than those of the primary GWAS, but the results in most of these regions were not supported by primary GWAS data (Figure S2). Thus, we proceeded to attempt to validate the primary GWAS findings based on the combined genotyped CAMP/LOCCS/LODO/Sepracor SNP results and HapMap Phase 2 imputed CARE and ACRN SNP results. The top five primary GWAS SNPs with P-value<1E-05 are in Table 2. Further details on these regions and all primary GWAS SNPs with P-value<1E-04 are in Tables S1 and S2. Further details on all 1000GP imputed GWAS SNPs with P-value<1E-05 are in Tables S3

**Table 1:** Characteristics of GWAS subjects.

	CAMP (n = 546)	Sepracor (n = 401)	LOCCS (n = 135)	LODO (n = 114)	CARE§ (n = 207)	ACRN¶ (n = 241)	SARP (n = 552)	DAG (n = 550)
Age	8.9 (2.1) [5, 13]	32.4 (14.0) [12, 80]	35.8 (14.7) [7, 67]	43.0 (15.0) [15, 76]	10.6 (2.9) [6, 18]	31.7 (10.5) [12, 64]	36.5 (13.1) [14, 68]	39.9 (12.6) [7, 75]
Male – N (%)	327 (60)	196 (49)	49 (36)	28 (25)	129 (62)	100 (41)	179 (32)	247 (45)
Wash-out Prior to BDR Test* - Weeks	4	6	4–6 on fluticasone	2	0–4	0–6	0	0–2
Albuterol puffs (90 µg/puff) for BDR Test	2	2	2	2	4	2 or 4	2 to 8	800 µg Salbutamol
Pre-BD FEV_1_	1.7 (0.5) [0.4, 3.3]	2.2 (0.5) [1.1, 3.6]	3.1 (0.8) [1.3, 5.1]	2.5 (0.7) [0.9, 4.9]	2.1 (0.7) [0.9, 5.5]	3.2 (0.8) [1.1, 5.6]	2.6 (0.9) [0.5, 15.9]	2.8 (0.9) [0.4, 5.9]
Pre-BD FEV_1_ % Predicted	94.0 (14.2) [51.0, 138.0]	57.7 (14.0) [23.3, 81.0]	87.4 (18.6) [38.6, 121.4]	76.8 (23.6) [30.3, 126.2]	98.5 (12.9) [69.6, 133.1]	87.0 (14.4) [35.0, 137.0]	76.7 (20.9) [5.9, 117.9]	80.1 (21.0) [11.6, 131.8]
BDR	10.8 (10.2) [−16.0, 60.0]	40.6 (20.9) [14.9, 140.5]	6.0 (6.3) [−11.1, 21.3]	6.0 (10.5) [−5.6, 60.3]	9.7 (8.4) [−16.7, 37.6]	11.6 (10.2) [−2.7, 73.9]	13.3 (16.1) [−6, 146]	17.5 (15.5) [−14.5, 131.2]
Genotyping Platform**	550Kv3 or 610	610	610	610	Affy 6.0	Affy 6.0	610	317K or 370K

Pre-BD = Pre-bronchodilator. SD = Standard deviation. For continuous variables, Mean (SD) and [Range] are shown.

*: Subjects were allowed to use rescue medication during wash-out period unless otherwise indicated.

**: 550Kv3, 610, 317K, and 370K refer to Illumina BeadChips.

**§:** CARE trials included: CLIC, PACT, MARS.

**¶:** ACRN trials included: BAGS, DICE, IMPACT, PRICE.

**Table 2:** Primary GWAS Top Results (SNPs with Combined P-values <1e-05).

				CLLS		CARE			ACRN				
SNP	CHR	BP	Minor Allele	MAF	P-value	MAF	P-value	Rsq	MAF	P-value	Rsq	Combined P-value	Gene
rs4452682	6	3360301	A	0.40	4.80E-05	0.44	0.021	0.89	0.46	0.018	0.79	3.20E-06	*SLC22A23*
rs295137	2	200858285	T	0.41	4.90E-05	0.41	1.10E-03	0.95	0.41	0.12	0.97	3.40E-06	*SPATS2L*
rs295114	2	200903847	T	0.43	3.50E-05	0.42	1.50E-03	0.96	0.42	0.42	0.99	6.00E-06	*SPATS2L*
rs10940113	5	66850598	C	0.37	1.00E-06	0.34	0.73	0.90	0.35	0.72	0.91	6.10E-06	
rs4328902	4	25348267	T	0.27	2.20E-05	0.27	0.38	0.73	0.26	0.026	0.78	6.10E-06	

CLLS = CAMP/LOCCS/LODO/Sepracor. CLLS used genotyped SNP data. CARE and ACRN used HapMap Phase 2 imputed data with Mach ratio of empirically observed dosage variance to the expected dosage variance (Rsq) values indicated.

### Replication of Primary GWAS Findings

**Figure 2:**
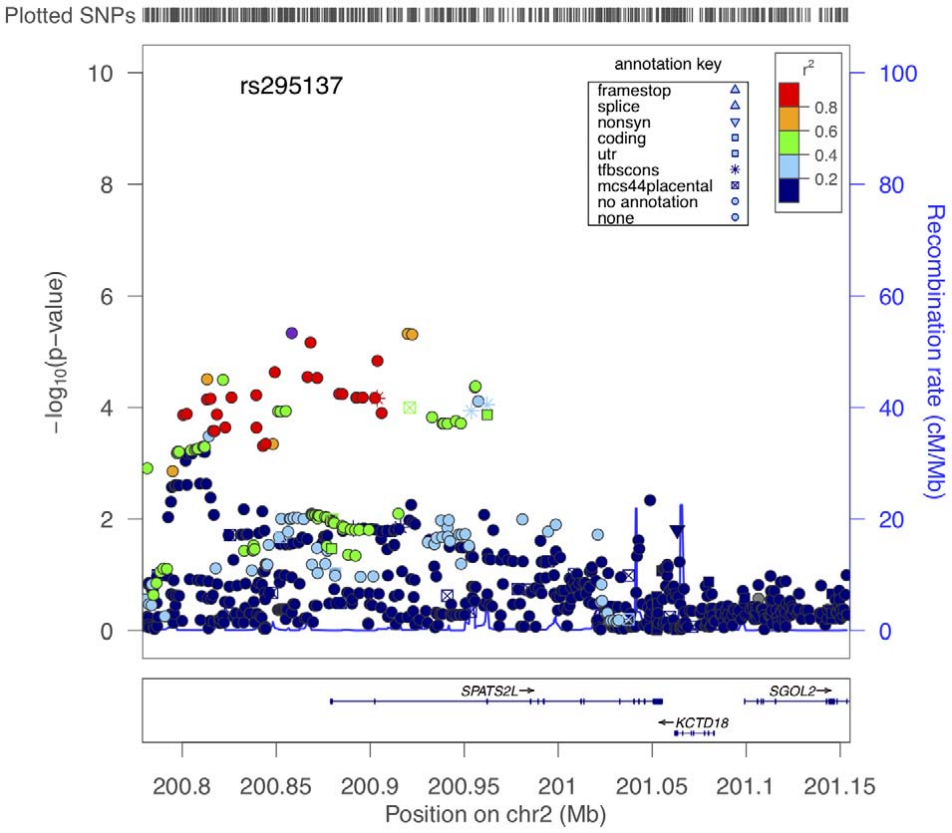
Region of association near *SPATS2L* SNPs to BDR. The x-axis denotes position along Chromosome 2. The y-axis denotes −Log_10_(P) corresponding to 1000GP imputed data P-values. LD between the SNP with the lowest P-value (rs295137) to each SNP in the plot is denoted in colors and was computed according to 1000GP June 2010 CEU data. Plot was created using LocusZoom [44].

**Figure 3:**
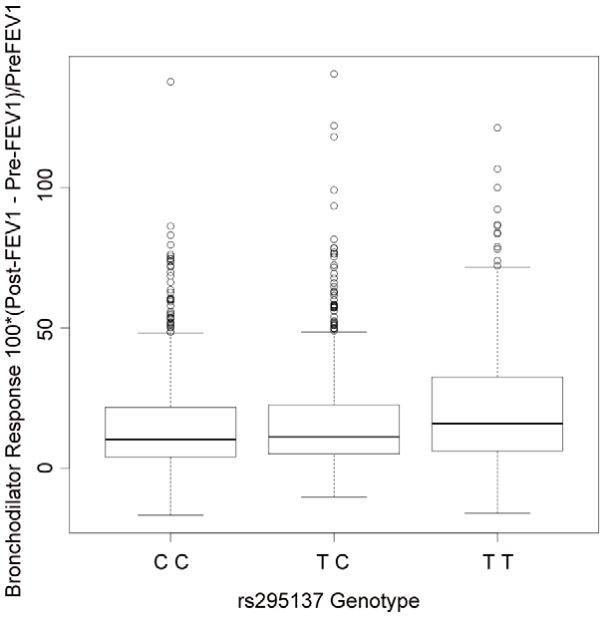
Summary of BDR by genotype of the SNP (rs295137) near *SPATS2L* with lowest P-value among all subjects in the primary populations. The TT genotype was associated with higher BDR (median 16.0; inter-quartile range (IQR) = [6.2, 32.4]), than the TC genotype (median 11.2; IQR = [5.2, 22.6]) or the CC genotype (median 10.3; IQR = [4.1, 21.7]).

We attempted to replicate in SARP all of the SNPs with primary GWAS P-values<1E-04 (Table S5). Three had nominally significant P-values (i.e. <0.05), and two of these associations supported the top 5 primary GWAS associations (Table 3). The lowest combined P-value for all primary GWAS plus SARP data was 7.7E-07 for rs295137. The region of BDR association spanning this SNP was in the 5′UTR region of *SPATS2L*, a gene of unknown *in vivo* function and paralog of *SPATS2* (Figure 2). The effect of the rs295137 genotype on BDR is shown in Figure 3, and a plot of the residuals of the linear regression fit of BDR while adjusting for age, sex, and height is shown in Figure S4. We sought further evidence of association for the two *SPATS2L* SNPs with lowest P-values in our primary GWAS in a second independent population: DAG. There was no evidence for association in this cohort (rs295137 P-value = 0.21; rs295114 P-value = 0.21), and combined P-values for these two SNPs across all cohorts were 9.7E-07 and 1.6E-06 (Table 3). To investigate whether our top combined association represented a biologically significant finding, we sought experimental evidence that *SPATS2L* was involved in bronchodilator response.

**Table 3:** Replication study of top SNPs in two independent populations (SARP and DAG).

	SARP		DAG		
SNP	MAF	P-value	MAF	P-value	Combined P-value*
rs4452682	0.50	0.43	-	-	1.3E-05
rs295137	0.41	0.044	0.40	0.21	9.7E-07
rs295114	0.43	0.043	0.42	0.21	1.6E-06
rs10940113	0.37	0.16	-	-	5.5E-06
rs4328902	0.34	0.91	-	-	2.8E-04

*: rs295137 and rs295114 include SARP and DAG P-values. Remaining SNPs include SARP only.

### *SPATS2L* mRNA Expression Changes when the β_2_-Agonist Pathway Is Modified in Human Airway Smooth Muscle Cells

We found one public gene expression array experiment (GSE13168) that would help to address the question of whether *SPATS2L* is differentially expressed in response to changes in the BDR pathway. We compared the levels of expression of two *SPATS2L* and one *SPATS2* probes in human airway smooth muscle (HASM) cells that stably expressed a PKA inhibitor vs. a GFP control at baseline and when stimulated with the pro-asthmatic cytokines interleukin-1β (IL1β), epidermal growth factor (EGF), or both. A trend of differential expression was observed for the *SPATS2* and one *SPATS2L* probes, but not a second *SPATS2L* probe (Figure S5). None of the comparisons met a Benjamini-Hochberg adjusted significance threshold, but nominally significant P-values were obtained for the *SPATS2* probe under all conditions and for one of the *SPATS2L* probes under the condition of EGF and IL1β stimulation (Table S6). According to the Gene Enrichment Profiler, the two *SPATS2L* probes are highly expressed in lung, and all three probes are highly expressed in smooth muscle, especially the *SPATS2* one. Overall, there are strikingly different tissue-specific expression patterns for each probe (Figure S6).

### Knockdown of *SPATS2L* mRNA Leads to Increased β_2_AR Levels

**Figure 4:**
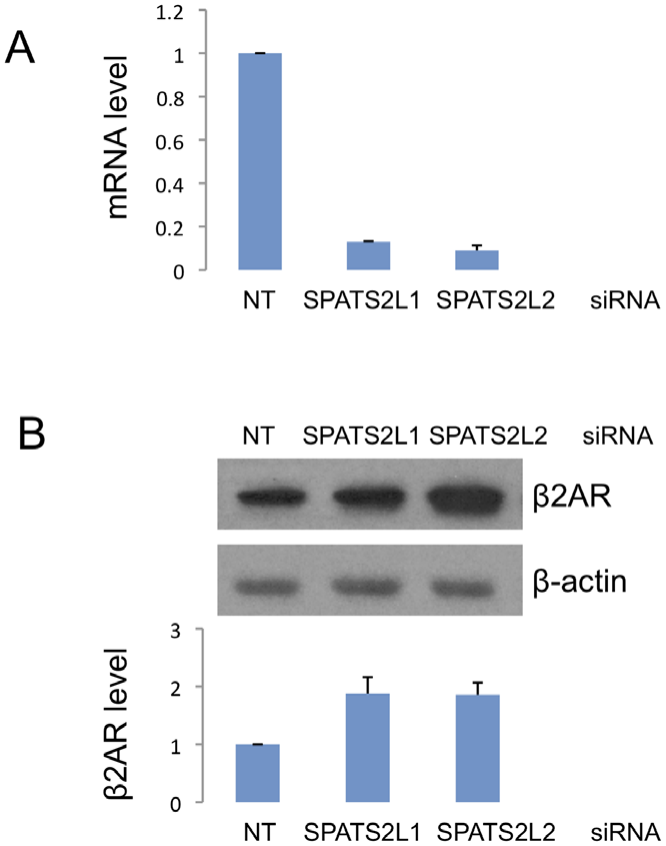
Effect of siRNA-mediated *SPATS2L* knockdown on β_2_-adrenergic receptor levels in HASM cells. (A) Knockdown efficiency of two *SPATS2L* siRNAs. Quantitative real-time PCR was done on RNAs extracted from HASM cells transfected with control non-targeting (NT) or *SPATS2L*-specific siRNAs. (B) Western blot analysis of β_2_AR protein in *SPATS2L* knockdown HASM cells. Three independent experiments (siRNA transfection and Western blot analysis) were done in HASM cells. The upper panel is a representative of the three Western blots. Quantification of the β_2_AR protein amount (normalized against the control β-actin protein) from three Western blots is shown in the lower panel.

We further investigated the involvement of *SPATS2L* in the β_2_-adrenergic response pathway by knocking down *SPATS2L* mRNA using two different small interfering RNAs (siRNA) and measuring subsequent changes in β_2_AR protein levels. The knockdown efficiency of the siRNAs was >80% reduction of *SPATS2L* mRNA as measured by qRT-PCR, and the corresponding increases in β_2_AR (normalized against the control β-actin protein) levels were 1.88- (SD 0.41) and 1.86- (SD 0.30) fold for the two *SPATS2L* siRNAs (Figure 4).

### Primary GWAS Results in Previously Identified BDR Candidate Genes

The association of SNPs with BDR at SNPs in/near genes (i.e. *ADRB2*, *ADCY9*, *CRHR2*, *ARG1*) previously reported as being associated with BDR was measured in our primary GWAS imputed data (Figure S7). Nominally significant (P-value<0.05) SNPs were found in *ADCY9*, *CRHR2*, and *ARG1*, but not in *ADRB2* (Tables S7 and S8). The SNPs with lowest P-values within 50 kb of these genes were: rs2531988 for *ADCY9* (3.2E-03), rs12533248 for *CRHR2* (0.029), and rs6929820 for *ARG1* (0.012).

## Discussion

In recent years, many GWAS that have successfully identified risk-modifying loci for a wide range of complex diseases have been published, but progress toward understanding how the loci and genes identified are functionally related to diseases has been slow [19]. The relationship of genes and gene variants to pharmacogenetic traits is often easier to test functionally than that for complex diseases because pharmacogenetic traits are more amenable to *in vitro* testing. However, compared to GWAS of complex diseases, GWAS of pharmacogenetic traits have been challenged by the relatively small size of drug clinical trials, which has caused many studies to be underpowered for obtaining genome-wide significant associations [20]. Nonetheless, successful pharmacogenetic GWAS have led to the identification of loci involved in modulating response to inhaled corticosteroids among asthma patients [21], warfarin dose [22], and lipid-lowering response to statins [23].

One of the difficulties specific to BDR GWAS is the complexity of the BDR phenotype. Regardless of how it is quantified, BDR is highly variable among asthma patients due to the time-dependent variation in baseline FEV_1_ and the influence of external environmental factors [24]. BDR can be quantified in various ways, with slightly varying resulting classification of patients as responders or non-responders. For our study, we selected the definition most widely utilized in clinical and human asthma research settings: percent change in baseline FEV_1_ following administration of a standard dose of short-acting inhaled β_2_-agonist [25]. We have attempted to control for the known relationship between baseline lung function and BDR [24], [26] by (1) selecting a definition of BDR that standardizes the change in FEV_1_ by dividing by baseline FEV_1_ and (2) by using age, sex, and height, which together account for a large portion of the variability in baseline lung function, as covariates in our statistical models. Because BDR tests are routinely performed during asthma clinical trials to use as inclusion criteria and to monitor outcomes among patients, we were able to utilize subjects from several diverse asthma clinical trials that were not specifically designed to study the pharmacogenetics of BDR. Most of these trials included a wash-out period that reduces modification of BDR due to concomitant medication administration, but LOCCS and some CARE and ACRN subjects were administered BDR tests at a time when they were not necessarily off of medications (Table 1). Subjects without a placebo washout, and especially those who were on ICS (e.g. LOCCS subjects), may be expected to have less BDR than those on placebo. The relationship of the magnitude of BDR to the various gene loci could therefore be blunted and show a less significant relationship than would be expected if all studies had incorporated a placebo washout.

In addition to variable washout periods, the cohorts had other significant differences in their design. Two trials consisted of children with asthma (i.e. CAMP, CARE), while the others consisted mostly of adults. The gender composition varied from 25% to 62% male. We attempted to control for age, gender and height, all of which are known to influence BDR, by including them as covariates in the association analysis. The mean and range of BDR also varied among trials. Of most significance, because the Sepracor trial used BDR greater than 15% as a criterion for inclusion, its subjects had markedly greater BDR than those of other trials. We attempted to control for this difference and any other trial specific differences among the cohorts that were pooled in the primary analysis by including trial as a covariate in the association model. There were additional differences among trials that were not taken into account. For example, SARP and Sepracor were composed of subjects with more severe asthma than those of other cohorts. Some ACRN, CARE, and SARP subjects were administered a different amount of albuterol during their BDR tests than those of CAMP, Sepracor, LOCCS, and LODO. DAG subjects were administered a different beta-agonist (i.e. Salbutamol) at a different concentration than that used with subjects of all other trials. DAG and SARP subjects were not participants of clinical trials, so there was greater heterogeneity of subjects within those cohorts. Despite the heterogeneity among trials, we utilized as many subjects as possible in an attempt to increase our statistical power to detect associations of SNPs with BDR. We reasoned that any associations detected despite the heterogeneity of the trial populations would be those most likely to generalize to all asthma patients. Another expected consequence of the trial heterogeneity is that our association results do not replicate in all cohorts. While having the largest number of subjects provides the greatest statistical power to detect statistically significant associations that are most generalizable across the clinical trials, we may be missing associations that are specific to the individual trials. For example, the subjects within clinical trials representing different ranges of asthma severity, age, and baseline characteristics may have genetic associations that are unique to subjects with their specific trial characteristics. The small sample size of each individual clinical trial makes detection of trial-specific associations more challenging. Despite the cohort heterogeneity, our meta-analysis identified a strong association that suggests a novel gene is involved in BDR.

Our top association was at SNP rs295137, with a combined P-value across all cohorts of 9.7E-07. This P-value does not meet conventional genome-wide significance thresholds (e.g. Bonferroni corrected minimally significant P-value would be 0.05/469,884 = 1.1E-07), but performing searches through public data sources and the fact that other pharmacogenetic GWAS have discovered biologically important results without genome-wide significant associations led us to pursue our top association further. The region of association surrounding rs295137 is in the 5′UTR of *SPATS2L* (Figure 2). This gene maps to chromosome 2 at 2q33.1, covering 176.78 kb from 201170592 to 201347368 (NCBI 37, August 2010). According to data gathered via the AceView [27] tool, *SPATS2L* is a complex locus that may have at least 30 spliced variants, its *in vivo* function is unknown, and it is a highly expressed gene in many tissues, with the greatest number of GenBank accessions belonging to lung. In gene-trap experiments in myoblasts, *SPATS2L* (a.k.a. *SGNP*) was found to be involved in ribosomal biogenesis and translational control in response to oxidative stress [28].

The availability of one public expression array experiment that utilized HASM cells expressing a PKA inhibitor (PKI) to modify the β_2_-adrenergic pathway allowed us to perform a preliminary search for evidence that *SPATS2L* may be involved in BDR. We found that a probe for *SPATS2*, the paralog of *SPATS2L*, was significantly differentially expressed in PKI vs. control cells at baseline and when stimulated with pro-inflammatory cytokines (EGF, IL1β, or both). One *SPAST2L* probe followed this trend but had a nominally significant P-value only under the condition of stimulation with both EGF and IL1β, while the other *SPATS2L* probe did not exhibit any changes. As illustrated in Figure S6, the tissue-specific expression patterns of the three probes varied widely. While all were expressed in smooth muscle, the *SPATS2* probe's relative expression in this tissue was markedly greater than that of the *SPATS2L* probes. Taken together, the expression patterns are consistent with tissue and isoform dependent changes in *SPATS2L* gene products. While the public dataset *SPATS2L* results were inconclusive based on the differences among probes, they suggested that *SPATS2L* expression may change when PKA is inhibited in HASM cells.

Knockdown of *SPATS2L* in HASM cells resulted in significantly increased β_2_AR protein levels, suggesting that *SPATS2L* may affect BDR by directly modulating β_2_AR protein expression. In HASM, β_2_-agonists exert their effects exclusively via the β_2_AR [6]. The relaxation of HASM occurs after the binding of β_2_-agonists to β_2_ARs via increased levels of cAMP followed by PKA activation. PKA activation leads to changes in gene transcription via activation of cAMP response element binding protein (CREB). Because β_2_ARs are the gateway to the effects of β_2_-agonists in HASM cells, modulations, such as *SPATS2L* inactivation, that increase the levels of β_2_ARs in HASM cells may lead to both greater relaxation in response to β_2_-agonists in the short term and greater differences in gene transcription in the longer term. Further study is needed to elucidate the precise mechanism by which *SPATS2L* regulates β_2_AR and consequently modifies BDR. Among our primary GWAS subjects, those whose *SPATS2L* SNP rs295137 has the TT genotype have greater BDR than those with CT or TT genotypes (median BDR 16.0 vs. 10.9). In one of the simplest scenarios, it is possible that the increased BDR among subjects with the TT genotype results from this genotype playing a direct role in decreasing transcription of *SPATS2L*, which in turn results in increased β_2_AR levels. Further work is required to understand how specific SNP associations in/near *SPATS2L* affect *SPATS2L* function and/or expression and how such effects impact β_2_AR signaling and BDR. Because the observed influence of our most strongly associated SNP genotype on BDR is relatively small (Figure 3), our current data do not support the development of any personalized therapeutics based solely on variants in/near *SPATS2L*.

In addition to studying top primary GWAS SNPs, we attempted to replicate findings from previous BDR candidate gene association studies. Specifically, we measured association between BDR and *ADRB2* [13], [14], *ADCY9* [15], *CRHR2* [16], and *ARG1* [17] variants. Notably, these previous findings are not entirely independent of those from the current GWAS: CAMP was a primary population utilized to identify associations in *ADRB2*, *ADCY9*, *CRHR2*, and *ARG1* in previous reports; LODO and Sepracor were replication populations in the *CRHR2*, and *ARG1* reports; and LOCCS was a replication population in the *ARG1* report. At a nominal significance level, we replicated gene-level associations for all of the candidate genes other than *ADRB2*. This gene, which encodes the β_2_AR, is the most studied gene related to BDR and SNPs and haplotypes in this gene have been related to decreased pulmonary function [29], response to β_2_-agonist treatment [30], an increased frequency of asthma exacerbations [31], and BDR [13], [14]. Initial reports of *ADRB2* associations were very promising and suggested that variants of this candidate gene would be reliable markers of BDR pharmacogenetics. However, a meta-analysis of 21 studies of *ADRB2* polymorphisms found that most of the positive associations identified in individual studies cannot be compared to findings in other studies due to different study designs, phenotypes considered and selective reporting, making the number of variants with true replications very small and questioning the utility of *ADRB2* polymorphisms for generalizable pharmacogenetic tests [32]. Our inability to find associations with *ADRB2* variants is consistent with the view that BDR genetics are complex: no individual SNPs or genes are responsible for a large proportion of BDR variability observed among all asthmatics. Our results suggest that genes other than the previously identified candidate genes are more strongly associated with BDR and that functional studies of these regions may yield important insights into BDR biology despite not having strong effects or generalizing to all populations.

In summary, a BDR GWAS among asthma patients from eight cohorts found that the most strongly associated SNP, rs295137, had a combined P-value of 9.7E-07. This association led us to *SPATS2L*, a gene of unknown *in vivo* function that we showed may be involved in BDR via the down-regulation of β_2_AR levels. Our results support the notion that there is promise in pursuing GWAS results that do not necessarily reach genome-wide significance and are an example of the way in which results from pharmacogenetic GWAS can be studied functionally.

## Materials and Methods

### Ethics Statement

Each study was approved by the Institutional Review Board of the corresponding institution, which ensured that all procedures followed were in accordance with the ethical standards of the responsible committee on human experimentation. Informed consent was obtained for all study participants.

### Subjects

The primary group of subjects consisted of 1,644 non-Hispanic white asthmatics from the following drug clinical trials: Childhood Asthma Management Program (CAMP) [33], Leukotriene Modifier or Corticosteroid Salmeterol study (LOCCS) [34], Effectiveness of Low Dose Theophylline as an Add-on Treatment in Asthma trial (LODO) [35], a medication trial conducted by Sepracor, Inc. [36], [37], and subsets of clinical trials within the Childhood Asthma Research and Education (CARE) network [38], and the Asthma Clinical Research Network (ACRN) [39] participating in the NHLBI SNP Health Association Resource (SHARe) Asthma Resource project (SHARP). Some basic characteristics of these cohorts are in Table 1 and further details are provided in Text S1. BDR tests were performed according to American Thoracic Society criteria with Albuterol as the β_2_-agonist [25], unless otherwise noted. Baseline BDR measures were utilized, and BDR was quantified as the percent change in FEV_1_ in response to administration of a β_2_-agonist [i.e. (post-BD FEV_1_ – pre-BD FEV_1_)/pre-BD FEV_1_].

### Genotyping and Quality Control

Genome-wide genotyping for CAMP subjects (n = 546) was performed on the HumanHap550 Genotyping BeadChip or Infinium HD Human610-Quad BeadChip by Illumina, Inc (San Diego, CA) at the Channing Laboratory. LOCCS (n = 135), LODO (n = 114), and Sepracor (n = 401) subjects were genotyped at the Riken Center for Genomic Medicine using the Infinium HD Human610-Quad BeadChip. CARE (n = 207) and ACRN (n = 241) subjects were genotyped on Affymetrix 6.0 genotyping chip by Affymetrix, Inc. (Santa Clara, CA). Data from those subjects genotyped using Illumina technologies was combined into a primary dataset with 469,884 overlapping SNPs having missingness <1%, passing HWE (P-value threshold of 1E-03), and having minor allele frequency (MAF)>0.05. EIGENSTRAT was used to identify 23 outliers (not included in counts above) based on being outside of six standard deviations of the top four principal components during five iterations [40]. The genomic inflation factor (λ_GC_) of the remaining 1,196 subjects was 1.002, demonstrating minimal population stratification. CARE and ACRN dataset quality control (QC) also included the removal of four related subjects (i.e. CARE siblings), SNPs with MAF<0.05, missingness >5%, or not passing HWE based on a threshold of 1E-03. The λ_GC_ for CARE and ACRN genotype data were 1.02 and 0.98, demonstrating minimal population stratification among subjects within each group. Comprehensive genotyping and QC measures are provided in Text S1.

### Statistical Analysis

Due to the poor overlap among SNPs genotyped on the Illumina and Affymetrix platforms, imputation of all SNPs available in HapMap Phase 2 Release 22 CEU data using the Markov Chain Haplotyping software (MaCH) [41] was performed for ACRN and CARE genotyped data. The primary GWAS consisted in the set of 469,884 SNPs that were successfully genotyped in those cohorts using Illumina arrays (i.e., CAMP/Sepracor/LOCCS/LODO) and imputed with HapMap Phase 2 data in those cohorts genotyped with Affymetrix arrays (i.e., ACRN and CARE) with a ratio of empirically observed dosage variance to the expected (binomial) dosage variance greater than 0.3, indicating good quality of imputation.

To expand the association results, imputation of all SNPs available in the June 2010 release of the 1000 Genome Project (1000GP) data using MaCH was performed for each of the three primary groups of genotype data. An overlapping set of 4,571,615 imputed SNPs had a MAF>0.05 and ratio of empirically observed dosage variance to the expected (binomial) dosage variance greater than 0.5, indicating good quality of imputation.

The association of SNPs with BDR was measured with a linear regression model as implemented in PLINK [42] in the three sets of data: 1) CAMP/Sepracor/LOCCS/LODO, 2) ACRN, 3) CARE. Association of imputed SNPs was carried out using dosage data. Covariates for the CAMP/Sepracor/LOCCS/LODO group included age, gender, height, and study. Covariates for the CARE and ACRN groups included age, gender, and height. To get the primary GWAS results, CARE and ACRN P-values were combined with those of the CAMP/Sepracor/LOCCS/LODO group by using Liptak's combined probability method [43] where hypothesis tests in CARE and ACRN had one-sided alternatives, based on the direction of the association in CAMP/Sepracor/LOCCS/LODO, so that SNPs with association tests in opposite directions would not produce inappropriately small P-values. The overall λ_GC_ was 1.002 in the primary set of GWAS results and 1.000 in the 1000GP imputed data GWAS.

Plots of association results near specific genes were created using LocusZoom with the hg18/1000 Genomes June 2010 CEU GenomeBuild/LD Population [44].

### Replication Studies

#### 1) Severe Asthma Research Program (SARP)

Replication of primary GWAS P-values was attempted in 501 European American subjects from SARP who were recruited to meet the American Thoracic Society (ATS) definition of mild to severe asthmatics, with enrichment for severe asthma [45]. Genotyping was performed on the Illumina Infinium HD Human 610-Quad Bead Chip at Wake Forest University. Tests of BDR association were performed using linear regression in R [46] with age, sex, and height as covariates.

#### 2) Dutch Asthma GWAS (DAG)

Replication of two SNPs near *SPATS2L* with lowest P-values in the primary GWAS was attempted in 550 DAG subjects with asthma and BDR data that were phenotyped in a single center (i.e, Beatrixoord, Haren, the Netherlands) [18], [47]. All patients refrained from bronchodilator use for at least 8 hours, and stopped inhaled steroids for 2 weeks before pulmonary function testing if clinically possible. Reversibility was assessed measuring spirometry before and 15 minutes after inhaling 800 µg of salbutamol. Genotyping was performed using the Hapmap 317K platform or Illumina 370 Duo Chip. Tests of BDR association were performed using linear regression, with age, gender and height as covariates using Predictive Analysis Software (PASW) version 18.0.

### Genome-Wide Gene Expression Data

The publicly available Gene Expression Omnibus (GEO) dataset, GSE13168, corresponding to an experiment in which human airway smooth muscle (HASM) cell cultures were generated from four donor trachea to test for the effects of glucocorticoids and PKA inhibition on the HASM transcriptome using the Affymetrix Human Genome U133A platform was used [48]. We tested for the involvement of our top primary GWAS gene in the β_2_-adrenergic pathway by comparing the differential expression of genes in cells stably expressing a PKA inhibitor (PKI) vs. control at baseline and in the presence of pro-inflammatory cytokines interleukin-1β (IL1β), epidermal growth factor (EGF), or both. The expression array contained two *SPATS2L* probes (i.e., 215617_at, 222154_s_at) and one *SPATS2* probe (i.e., 218324_s_at). The probe for the paralog of *SPATS2L* was included to account for the possibility of non-specific binding of *SPATS2L* mRNA to the *SPATS2* probe. Analyses were conducted in R [46]. Pre-processing of raw signal intensities was performed with RMA [49] and differential expression was quantified using the limma package [50]. Tissue-specific expression of these probes was assessed using 557 microarrays from 126 human normal primary tissues in the Gene Enrichment Profiler [51].

### *SPATS2L* siRNA Knockdown and β2AR Western Blotting Analysis

Primary HASM cells were isolated from aborted lung transplant donors with no chronic illness. The tissue was obtained from the National Disease Resource Interchange (NDRI) and their use approved by the University of Pennsylvania IRB. HASM cell cultivation and characterization were described previously [52], [53]. Passages 4 to 7 HASM cells maintained in Ham's F12 medium supplemented with 10% FBS were used in all experiments. 2×10^5^ HASM cells were grown overnight and then transfected with 50 nM siRNA by using DharmaFECT 1 reagent (Thermo Scientific, Lafayette, CO, USA). About 72 h post transfection, cells were washed with PBS and lysed with NP-40 lysing buffer (50 mM Tris-HCl pH7.5, 150 mM NaCl, 0.5% Nonidet P-40) containing protease inhibitor cocktail (Roche Diagnostics Corporation, Indianapolis, IN, USA). Protein samples were denatured 10 min at 50°C, separated on NuPAGE 4–12% Bis-Tris gels (Invitrogen, Grand Island, NY, USA) and transferred to PVDF membranes (Bio-Rad Laboratories, Hercules, CA, USA). Immunoblot signals were developed using SuperSignal West Pico (Pierce Protein Research Products, Thermo Fisher Scientific, Rockford, IL, USA) and quantified by ImageJ software. Non-targeting control siRNA, *SPATS2L*-specific siRNA 1 (sense 5′- guc agu cca uug auu guc u(dT)(dT)-3′, antisense 5′- aga caa uca aug gac uga c(dT)(dT) -3′) and *SPATS2L*-specific siRNA 2 (sense 5′-caa ccu gug uug uag cag u(dT)(dT)-3′, antisense 5′- acu gcu aca aca cag guu g(dT)(dT) -3′) were obtained from Sigma-Aldrich (Mission siRNA; St. Louis, MO, USA). Antibodies for β_2_AR (H20) and β-actin were from Santa Cruz Biotechnology, Inc. (Santa Cruz, CA, USA). Experiments were performed in triplicate.

## Supporting Information

Figure S1 Quantile–quantile plot. Comparison of primary GWAS P-values to those expected for a null distribution. There is little evidence of deviation of measures at the tail, obscuring the distinction among SNPs having low p-values representing true associations vs. those SNPs having low p-values by chance.
(TIFF)

Figure S2 Manhattan plot. The x-axis denotes position along each chromosome. The y-axis denotes −Log_10_(P) corresponding to association P-values. The 1000GP imputed results are in light blue and green. The primary GWAS results are in dark blue and green. Some of the lowest imputed P-values did not have corresponding primary GWAS p-values, while other regions with low P-values contained both primary and imputed GWAS results. We prioritized regions based on primary GWAS results.
(TIFF)

Figure S3 P-value comparison between Primary GWAS vs. 1000GP imputed results, for those SNPs contained in both datasets. The results are highly correlated (r^2^ = 0.99).
(TIFF)

Figure S4 Residuals of linear regression fit of BDR ∼ age + sex + height vs. rs295137 genotypes.
(TIFF)

Figure S5 Distribution of normalized signal intensities across different experimental conditions for two *SPATS2L* and one *SPATS2* probes corresponding to a subset of GSE13168 arrays where the effects of protein kinase A inhibition (PKI) in human airway smooth muscle cells was assessed at baseline and following stimulation with epidermal growth factor (EGF), interleukin 1 beta (IL1b), or both. Corresponding P-values and log-Fold Change differences are in Table S4. A) *SPATS2* probe 218324_s_at, B) *SPATS2L* probe 215617_at, C) *SPATS2L* probe 222154_s_at.
(TIF)

Figure S6 Tissue-specific gene expression patterns of Affy U133A probes for *SPATS2* and *SPATS2L* (a.k.a. *LOC26010*).
(TIFF)

Figure S7 Plots of imputed association results near previously identified BDR candidate genes. A) *ADCY9*, B) *ADRB2* C) *ARG1*, D) *CRHR2*.
(TIFF)

Table S1 Primary GWAS SNP details for SNPs with Primary GWAS P<1E-04.
(DOCX)

Table S2 Primary GWAS P-values and Beta coefficients for SNPs with Primary GWAS P<1E-04. CARE and ACRN P-values are 1-sided based on the direction in CAMP/LOCCS/LODO/Sepracor. CAMP/LOCCS/LODO/Sepracor reference allele was minor allele as shown in Table 1. SE = Standard Error for corresponding Beta coefficient. Rsq = MACH R-squared value for imputed SNP. Combined P-values were obtained using the Liptak method, with weights proportional to population size.
(DOCX)

Table S3 Primary GWAS 1000GP Imputed SNP details for SNPs with P<1E-05. MAF = Minor Allele Frequency. Rsq = MACH R-squared value for imputed SNP.
(DOCX)

Table S4 Primary GWAS 1000GP Imputed SNP P-values and Beta coefficients for SNPs with P<1E-05. CARE and ACRN P-values are 1-sided based on the direction in CAMP/LOCCS/LODO/Sepracor. SE = Standard Error for corresponding Beta coefficient. Combined P-values were obtained using the Liptak method, with weights proportional to population size.
(DOCX)

Table S5 SARP Replication Results for SNPs with Primary GWAS P<1E-04. P-values are 1-sided based on the direction in CAMP/LOCCS/LODO/Sepracor. MAF = minor allele frequency.
(DOCX)

Table S6 Unadjusted P-values and log fold-change quantifying differential expression of *SPATS2L* and *SPATS2* probes for GEO dataset GSE13168, in which human ASM cell lines expressing a PKA inhibitor vs. a GFP control were compared at baseline and when stimulated with IL1b, EGF, or both.
(DOCX)

Table S7 Primary GWAS 1000GP Imputed SNP details for SNPs that have nominally significant p-values (<0.05) in, or within 50,000 KB of, genes (i.e. *ADRB2*, *ADCY9*, *CRHR2*, *ARG1*) previously identified as being associated with BDR. There were no such SNPs near *ADRB2*. MAF = Minor Allele Frequency. Rsq = MACH R-squared value for imputed SNP.
(DOCX)

Table S8 Primary GWAS 1000GP Imputed SNP P-values and Beta coefficients for SNPs that have nominally significant p-values (<0.05), and are in, or within 50,000 KB of, genes (i.e. *ADRB2*, *ADCY9*, *CRHR2*, *ARG1*) previously identified as being associated with BDR. There were no such SNPs near *ADRB2*. CARE and ACRN P-values are 1-sided based on the direction in CAMP/LOCCS/LODO/Sepracor. SE = Standard Error for corresponding Beta coefficient. Combined P-values were obtained using the Liptak method, with weights proportional to population size.
(DOCX)

Text S1 Detailed subject characteristics and genotype data quality control.
(DOC)
