# Supplementary figures and images for: Genome-Wide Association Analysis in Asthma Subjects Identifies SPATS2L as a Novel Bronchodilator Response Gene
Source: PLoS Genet. 2012 Jul 5;8(7):e1002824. doi: 10.1371/journal.pgen.1002824 (PMC3390407; doi:10.1371/journal.pgen.1002824)

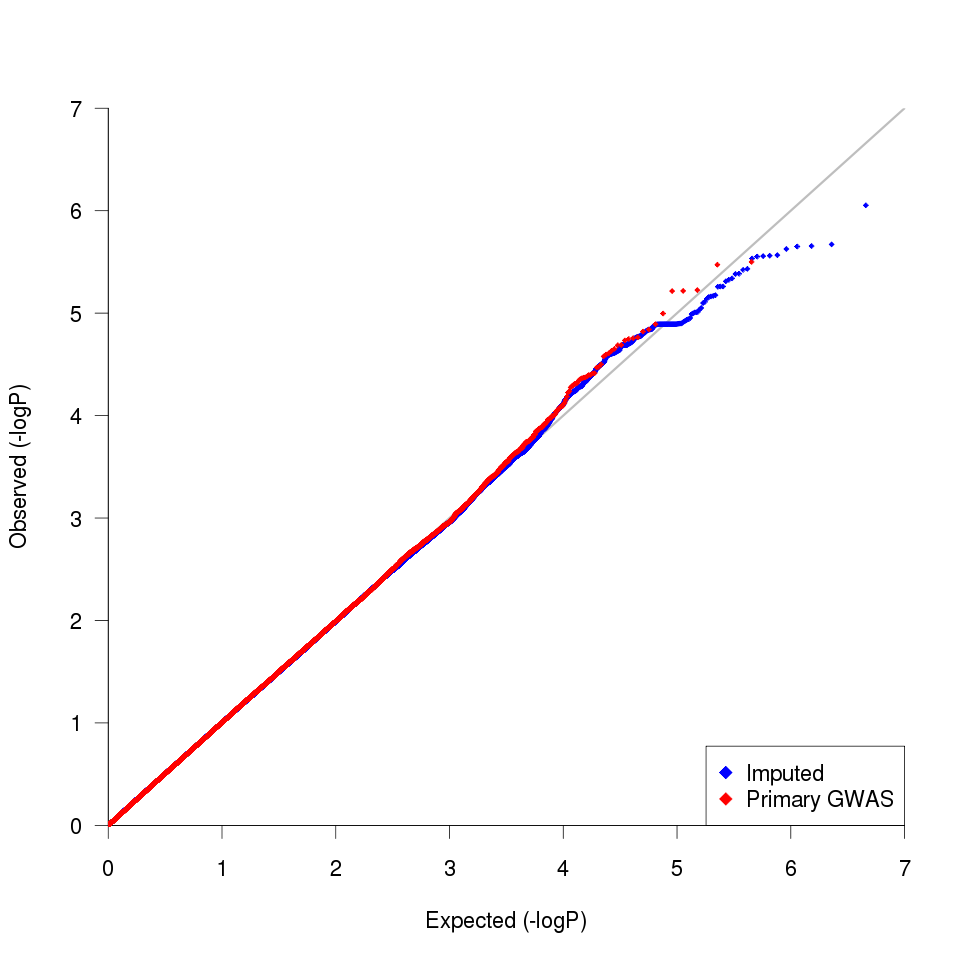

Supplement: Figure S1 — Quantile–quantile plot. Comparison of primary GWAS P-values to those expected for a null distribution. There is little evidence of deviation of measures at the tail, obscuring the distinction among SNPs having low p-values representing true associations vs. those SNPs having low p-values by chance. (TIFF) [file pgen.1002824.s001.tif]

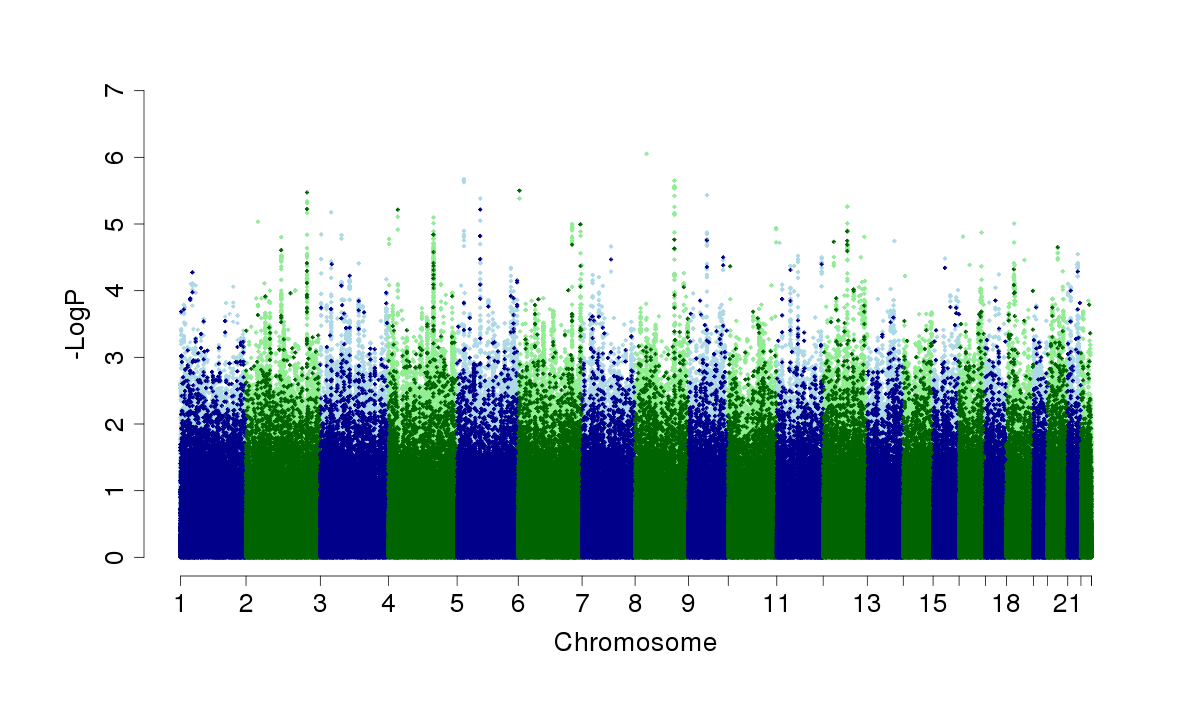

Supplement: Figure S2 — Manhattan plot. The x-axis denotes position along each chromosome. The y-axis denotes −Log10(P) corresponding to association P-values. The 1000GP imputed results are in light blue and green. The primary GWAS results are in dark blue and green. Some of the lowest imputed P-values did not have corresponding primary GWAS p-values, while other regions with low P-values contained both primary and imputed GWAS results. We prioritized regions based on primary GWAS results. (TIFF) [file pgen.1002824.s002.tif]

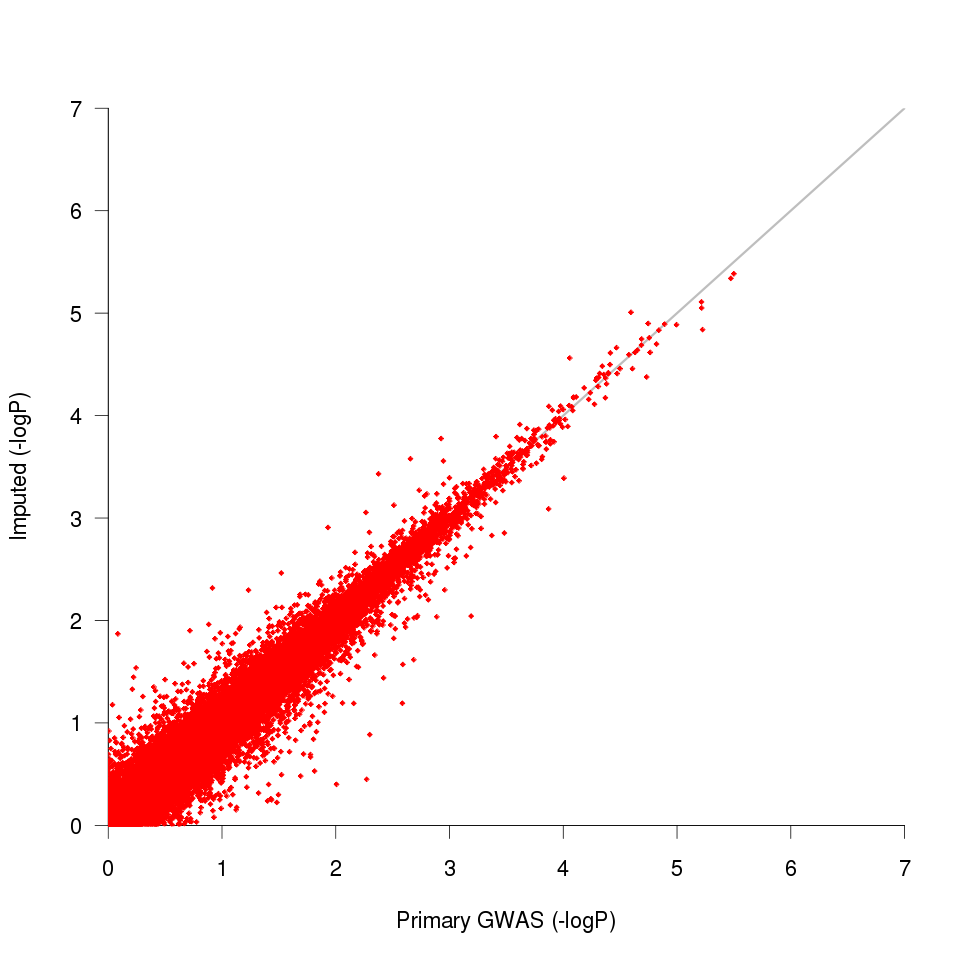

Supplement: Figure S3 — P-value comparison between Primary GWAS vs. 1000GP imputed results, for those SNPs contained in both datasets. The results are highly correlated (r2 = 0.99). (TIFF) [file pgen.1002824.s003.tif]

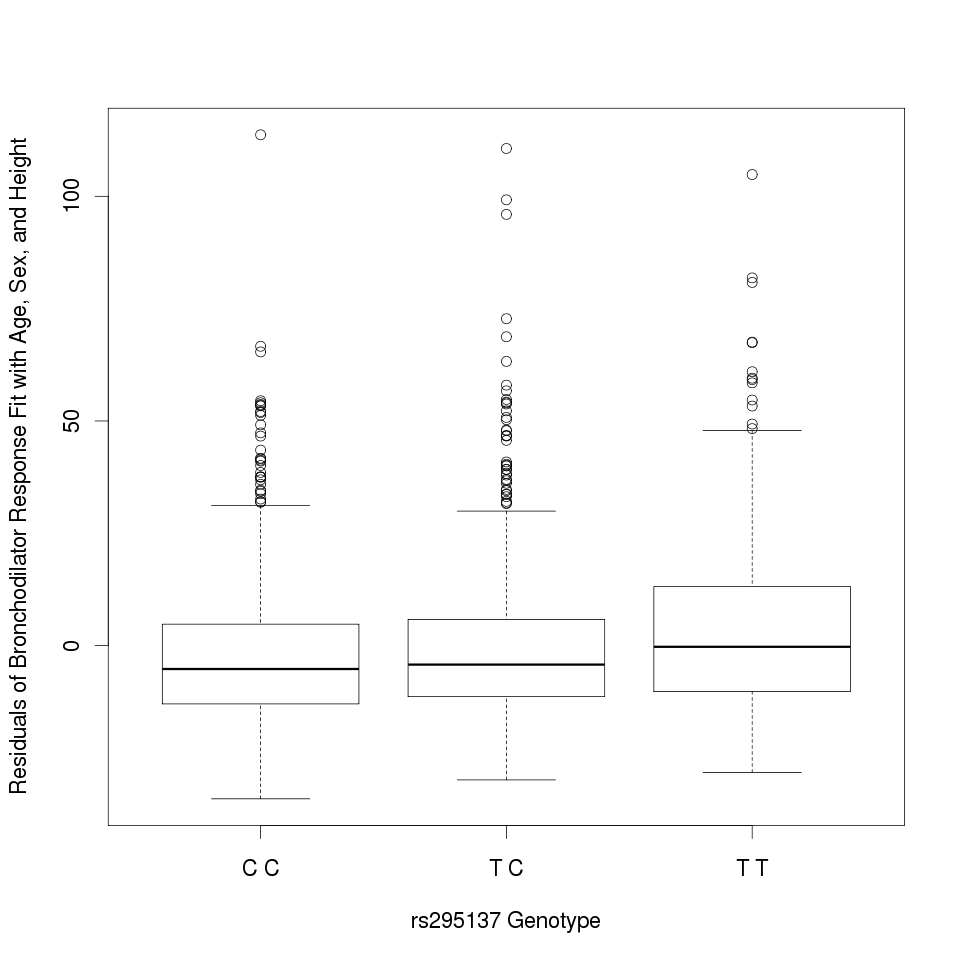

Supplement: Figure S4 — Residuals of linear regression fit of BDR ∼ age + sex + height vs. rs295137 genotypes. (TIFF) [file pgen.1002824.s004.tif]

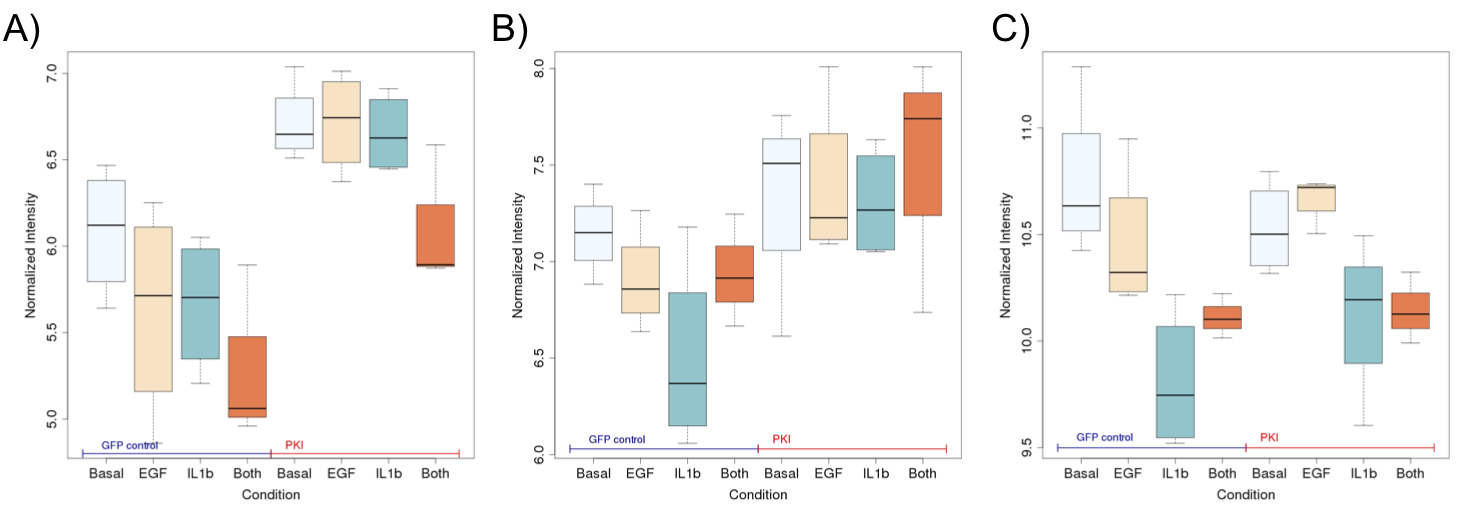

Supplement: Figure S5 — Distribution of normalized signal intensities across different experimental conditions for two SPATS2L and one SPATS2 probes corresponding to a subset of GSE13168 arrays where the effects of protein kinase A inhibition (PKI) in human airway smooth muscle cells was assessed at baseline and following stimulation with epidermal growth factor (EGF), interleukin 1 beta (IL1b), or both. Corresponding P-values and log-Fold Change differences are in Table S4. A) SPATS2 probe 218324_s_at, B) SPATS2L probe 215617_at, C) SPATS2L probe 222154_s_at. (TIF) [file pgen.1002824.s005.tif]

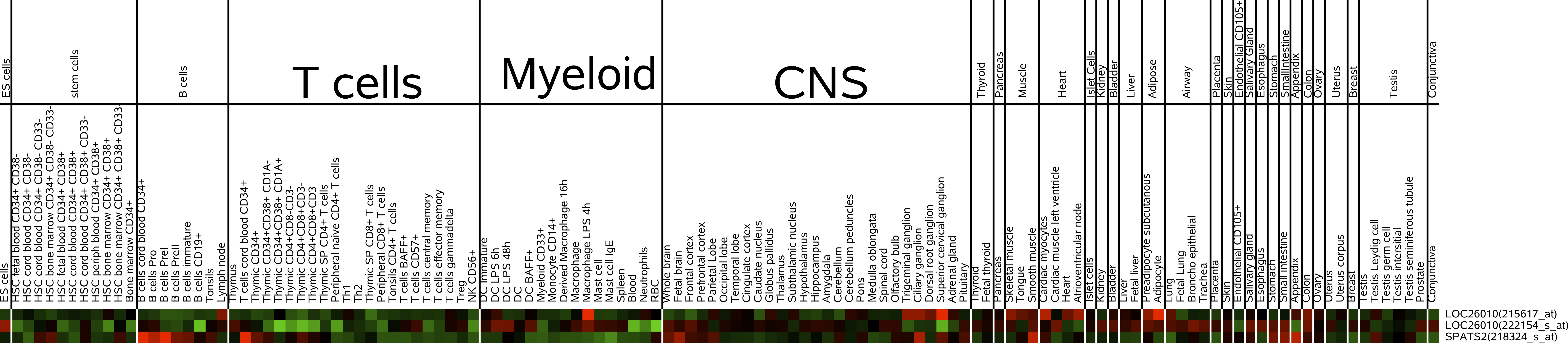

Supplement: Figure S6 — Tissue-specific gene expression patterns of Affy U133A probes for SPATS2 and SPATS2L (a.k.a. LOC26010). (TIFF) [file pgen.1002824.s006.tif]

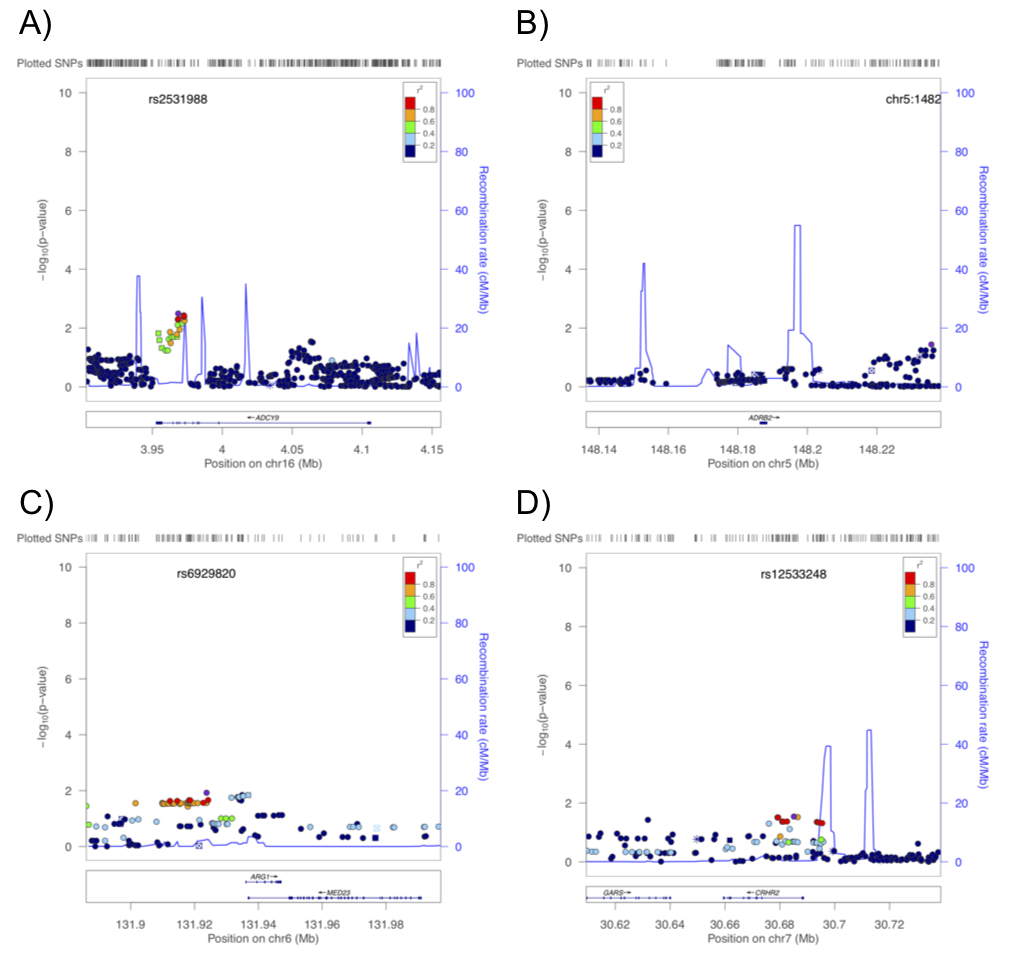

Supplement: Figure S7 — Plots of imputed association results near previously identified BDR candidate genes. A) ADCY9, B) ADRB2 C) ARG1, D) CRHR2. (TIFF) [file pgen.1002824.s007.tif]
