# Supplementary material for: Genome-Wide Association Analysis in Asthma Subjects Identifies SPATS2L as a Novel Bronchodilator Response Gene
Source: PLoS Genet. 2012 Jul 5;8(7):e1002824. doi: 10.1371/journal.pgen.1002824 (PMC3390407; doi:10.1371/journal.pgen.1002824)
Supplement: Table S1 — Primary GWAS SNP details for SNPs with Primary GWAS P<1E-04. (DOCX) [file pgen.1002824.s008.docx]

|  |  | |  | |  |  | Minor Allele Frequency (MAF) | | |
| --- | --- | --- | --- | --- | --- | --- | --- | --- | --- |
| SNP | | CHR | | BP | Minor Allele | Major Allele | CAMP/LOCCS/  LODO/Sepracor | CARE | ACRN |
| rs4452682 | | 6 | | 3360301 | A | G | 0.40 | 0.44 | 0.46 |
| rs295137 | | 2 | | 200858285 | T | C | 0.41 | 0.41 | 0.41 |
| rs295114 | | 2 | | 200903847 | T | C | 0.43 | 0.42 | 0.42 |
| rs10940113 | | 5 | | 66850598 | C | T | 0.37 | 0.34 | 0.35 |
| rs4328902 | | 4 | | 25348267 | T | C | 0.27 | 0.27 | 0.26 |
| rs2178806 | | 6 | | 167138752 | T | C | 0.15 | 0.12 | 0.15 |
| rs12306576 | | 12 | | 72808323 | G | A | 0.12 | 0.13 | 0.14 |
| rs10518485 | | 4 | | 127216424 | T | C | 0.21 | 0.21 | 0.21 |
| rs4976079 | | 5 | | 66842798 | T | C | 0.49 | 0.47 | 0.46 |
| rs12682157 | | 8 | | 107400829 | G | A | 0.22 | 0.21 | 0.22 |
| rs4581121 | | 9 | | 75000213 | C | T | 0.25 | 0.26 | 0.24 |
| rs7305291 | | 12 | | 72731874 | T | C | 0.12 | 0.13 | 0.14 |
| rs7958129 | | 12 | | 29989110 | T | G | 0.23 | 0.18 | 0.19 |
| rs17535206 | | 6 | | 147132767 | G | T | 0.06 | 0.07 | 0.06 |
| rs11179922 | | 12 | | 72791798 | C | T | 0.11 | 0.10 | 0.12 |
| rs6061043 | | 20 | | 29990726 | C | T | 0.43 | 0.41 | 0.41 |
| rs7013315 | | 8 | | 107396076 | G | A | 0.22 | 0.21 | 0.22 |
| rs10211073 | | 2 | | 113173520 | G | A | 0.49 | 0.48 | 0.50 |
| rs11179933 | | 12 | | 72815097 | T | C | 0.17 | 0.18 | 0.19 |
| rs1320269 | | 4 | | 127192494 | G | T | 0.20 | 0.21 | 0.20 |
| rs1889318 | | 9 | | 118882872 | C | T | 0.42 | 0.40 | 0.39 |
| rs4976096 | | 5 | | 66947989 | T | C | 0.26 | 0.24 | 0.24 |
| rs2367910 | | 7 | | 81623979 | A | C | 0.12 | 0.12 | 0.10 |
| rs6534528 | | 4 | | 127187429 | G | A | 0.20 | 0.21 | 0.20 |
| rs17531061 | | 2 | | 200821697 | C | A | 0.28 | 0.26 | 0.26 |
| rs12496948 | | 3 | | 26660543 | A | G | 0.24 | 0.24 | 0.25 |
| rs1857922 | | 11 | | 131025578 | T | C | 0.36 | 0.37 | 0.37 |
| rs1334086 | | 9 | | 118890634 | A | G | 0.44 | 0.42 | 0.41 |
| rs17010666 | | 4 | | 127226033 | C | A | 0.20 | 0.22 | 0.21 |
| rs17590608 | | 6 | | 167106014 | C | T | 0.26 | 0.24 | 0.26 |
| rs11252394 | | 10 | | 4230870 | A | G | 0.08 | 0.08 | 0.07 |
| rs10781126 | | 9 | | 74982121 | A | G | 0.31 | 0.30 | 0.30 |
| rs4775229 | | 15 | | 58081006 | C | A | 0.11 | 0.12 | 0.08 |
| rs11564299 | | 18 | | 24014026 | G | A | 0.23 | 0.22 | 0.19 |
| rs1348879 | | 11 | | 35550461 | A | G | 0.16 | 0.17 | 0.16 |
| rs7685518 | | 4 | | 127261004 | G | A | 0.23 | 0.24 | 0.24 |
| rs7598349 | | 2 | | 200955724 | G | A | 0.46 | 0.44 | 0.46 |
| rs2242930 | | 21 | | 39322828 | A | G | 0.28 | 0.27 | 0.31 |
| rs6661901 | | 1 | | 48968647 | G | A | 0.15 | 0.14 | 0.16 |
| rs7667104 | | 4 | | 127252636 | G | A | 0.23 | 0.25 | 0.24 |
| rs7644264 | | 3 | | 82094370 | T | C | 0.13 | 0.14 | 0.15 |
| rs7677190 | | 4 | | 127255830 | G | T | 0.23 | 0.24 | 0.24 |
| rs7730346 | | 5 | | 177306131 | T | G | 0.10 | 0.06 | 0.10 |
| rs4958980 | | 5 | | 177290570 | A | G | 0.10 | 0.06 | 0.10 |
| rs159320 | | 2 | | 200896020 | G | A | 0.46 | 0.46 | 0.44 |
| rs6414806 | | 5 | | 66856030 | T | C | 0.47 | 0.47 | 0.46 |
| rs565659 | | 4 | | 127285342 | C | A | 0.27 | 0.28 | 0.29 |
| rs11083252 | | 18 | | 23980721 | A | G | 0.23 | 0.24 | 0.22 |
| rs9838009 | | 3 | | 59240355 | T | G | 0.14 | 0.16 | 0.15 |
| rs7754623 | | 6 | | 167135374 | A | C | 0.13 | 0.11 | 0.13 |
| rs10097529 | | 8 | | 133773894 | A | C | 0.14 | 0.14 | 0.14 |
| rs1586150 | | 4 | | 127294208 | T | C | 0.23 | 0.24 | 0.24 |
| rs4146626 | | 12 | | 92885658 | G | A | 0.37 | 0.41 | 0.38 |
| rs2811686 | | 6 | | 134411117 | G | T | 0.23 | 0.24 | 0.24 |
| rs2826840 | | 21 | | 21731948 | G | A | 0.15 | 0.17 | 0.14 |
