# Supplementary material for: Genome-Wide Association Analysis in Asthma Subjects Identifies SPATS2L as a Novel Bronchodilator Response Gene
Source: PLoS Genet. 2012 Jul 5;8(7):e1002824. doi: 10.1371/journal.pgen.1002824 (PMC3390407; doi:10.1371/journal.pgen.1002824)
Supplement: Table S2 — Primary GWAS P-values and Beta coefficients for SNPs with Primary GWAS P<1E-04. CARE and ACRN P-values are 1-sided based on the direction in CAMP/LOCCS/LODO/Sepracor. CAMP/LOCCS/LODO/Sepracor reference allele was minor allele as shown in Table 1. SE = Standard Error for corresponding Beta coefficient. Rsq = MACH R-squared value for imputed SNP. Combined P-values were obtained using the Liptak method, with weights proportional to population size. (DOCX) [file pgen.1002824.s009.docx]

|  | CAMP/LOCCS/  LODO/Sepracor | | | CARE/ACRN | CARE | | | | | | | ACRN | | | | |  | |
| --- | --- | --- | --- | --- | --- | --- | --- | --- | --- | --- | --- | --- | --- | --- | --- | --- | --- | --- |
| SNP | Beta | SE | P-value | Reference Allele | Beta | SE | | Rsq | | P-value | | Beta | SE | Rsq | P-value | | Combined P-value | |
| rs4452682 | -3.00 | 0.74 | 4.8E-05 | A | -1.84 | | 0.90 | | 0.89 | | 2.1E-02 | -2.17 | 1.03 | 0.79 | 1.8E-02 | 3.2E-06 | |  |
| rs295137 | 2.95 | 0.72 | 4.9E-05 | C | -2.55 | | 0.82 | | 0.95 | | 1.1E-03 | -1.13 | 0.98 | 0.97 | 1.2E-01 | 3.4E-06 | |  |
| rs295114 | 3.01 | 0.73 | 3.5E-05 | C | -2.43 | | 0.81 | | 0.96 | | 1.5E-03 | -0.19 | 0.96 | 0.99 | 4.2E-01 | 6.0E-06 | |  |
| rs10940113 | -3.66 | 0.74 | 1.0E-06 | C | 0.54 | | 0.88 | | 0.90 | | 7.3E-01 | 0.59 | 0.98 | 0.91 | 7.2E-01 | 6.1E-06 | |  |
| rs4328902 | -3.48 | 0.82 | 2.2E-05 | C | 0.34 | | 1.16 | | 0.73 | | 3.8E-01 | 2.35 | 1.20 | 0.78 | 2.6E-02 | 6.1E-06 | |  |
| rs2178806 | 4.39 | 1.01 | 1.5E-05 | C | 0.96 | | 1.26 | | 0.89 | | 7.8E-01 | -2.46 | 1.34 | 0.95 | 3.4E-02 | 1.0E-05 | |  |
| rs12306576 | 4.74 | 1.10 | 1.8E-05 | A | -1.12 | | 1.23 | | 0.97 | | 1.8E-01 | -0.45 | 1.38 | 1.00 | 3.7E-01 | 1.3E-05 | |  |
| rs10518485 | -3.93 | 0.90 | 1.5E-05 | C | 0.36 | | 1.03 | | 0.95 | | 3.6E-01 | 0.56 | 1.13 | 0.97 | 3.1E-01 | 1.4E-05 | |  |
| rs4976079 | -3.26 | 0.73 | 7.9E-06 | C | -0.12 | | 0.94 | | 0.76 | | 5.5E-01 | 0.10 | 1.02 | 0.81 | 4.6E-01 | 1.5E-05 | |  |
| rs12682157 | -3.53 | 0.87 | 5.4E-05 | A | 0.25 | | 1.05 | | 0.96 | | 4.1E-01 | 2.10 | 1.13 | 1.00 | 3.3E-02 | 1.7E-05 | |  |
| rs4581121 | 3.35 | 0.82 | 5.0E-05 | C | -0.54 | | 1.00 | | 0.96 | | 7.0E-01 | 2.71 | 1.12 | 1.00 | 8.2E-03 | 1.8E-05 | |  |
| rs7305291 | 4.68 | 1.11 | 2.5E-05 | C | -1.12 | | 1.23 | | 0.97 | | 1.8E-01 | -0.45 | 1.38 | 1.00 | 3.7E-01 | 1.8E-05 | |  |
| rs7958129 | -3.34 | 0.87 | 1.2E-04 | G | 1.96 | | 1.22 | | 0.80 | | 5.5E-02 | 2.08 | 1.31 | 0.83 | 5.7E-02 | 1.8E-05 | |  |
| rs17535206 | 6.22 | 1.58 | 8.8E-05 | G | 3.33 | | 1.69 | | 0.95 | | 2.5E-02 | 1.50 | 1.95 | 1.00 | 2.2E-01 | 2.0E-05 | |  |
| rs11179922 | 5.15 | 1.19 | 1.6E-05 | C | 0.37 | | 1.31 | | 0.98 | | 3.9E-01 | 0.30 | 1.44 | 1.00 | 4.2E-01 | 2.1E-05 | |  |
| rs6061043 | 3.06 | 0.71 | 1.9E-05 | C | -0.61 | | 0.82 | | 0.95 | | 7.7E-01 | 1.08 | 0.94 | 0.99 | 1.3E-01 | 2.2E-05 | |  |
| rs7013315 | -3.46 | 0.87 | 7.2E-05 | A | 0.25 | | 1.05 | | 0.96 | | 4.1E-01 | 2.10 | 1.13 | 1.00 | 3.3E-02 | 2.3E-05 | |  |
| rs10211073 | -2.97 | 0.71 | 3.4E-05 | A | 0.31 | | 0.80 | | 0.95 | | 3.5E-01 | 0.69 | 0.93 | 0.99 | 2.3E-01 | 2.5E-05 | |  |
| rs11179933 | 3.92 | 0.98 | 7.3E-05 | C | -0.94 | | 1.07 | | 0.97 | | 1.9E-01 | -1.34 | 1.11 | 1.00 | 1.1E-01 | 2.5E-05 | |  |
| rs1320269 | -3.85 | 0.91 | 2.7E-05 | G | -0.22 | | 1.05 | | 0.95 | | 4.2E-01 | -0.59 | 1.12 | 0.99 | 3.0E-01 | 2.6E-05 | |  |
| rs1889318 | 2.66 | 0.73 | 3.0E-04 | C | 2.02 | | 0.86 | | 0.96 | | 9.8E-03 | 1.57 | 1.04 | 1.00 | 6.6E-02 | 3.2E-05 | |  |
| rs4976096 | -3.83 | 0.83 | 4.6E-06 | C | -0.02 | | 0.99 | | 0.96 | | 5.1E-01 | -1.69 | 1.10 | 1.00 | 9.4E-01 | 3.4E-05 | |  |
| rs2367910 | 4.53 | 1.12 | 6.1E-05 | A | 1.15 | | 1.31 | | 0.97 | | 1.9E-01 | 0.92 | 1.46 | 1.00 | 2.7E-01 | 3.4E-05 | |  |
| rs6534528 | -3.77 | 0.92 | 4.2E-05 | A | 0.23 | | 1.05 | | 0.95 | | 4.2E-01 | 0.66 | 1.12 | 0.99 | 2.8E-01 | 3.9E-05 | |  |
| rs17531061 | 3.08 | 0.81 | 1.5E-04 | A | -2.38 | | 0.93 | | 0.95 | | 5.5E-03 | -0.15 | 1.10 | 0.98 | 4.5E-01 | 3.9E-05 | |  |
| rs12496948 | -3.31 | 0.84 | 8.4E-05 | A | -0.82 | | 0.97 | | 0.91 | | 2.0E-01 | -0.93 | 1.10 | 0.96 | 2.0E-01 | 4.0E-05 | |  |
| rs1857922 | -3.41 | 0.75 | 6.9E-06 | C | 0.13 | | 0.88 | | 0.96 | | 4.4E-01 | -1.38 | 0.95 | 1.00 | 9.3E-01 | 4.0E-05 | |  |
| rs1334086 | 2.41 | 0.72 | 8.6E-04 | A | 2.46 | | 0.88 | | 0.96 | | 2.7E-03 | 2.43 | 1.07 | 0.99 | 1.2E-02 | 4.2E-05 | |  |
| rs17010666 | -3.72 | 0.92 | 5.4E-05 | A | 0.55 | | 1.01 | | 0.96 | | 2.9E-01 | 0.57 | 1.14 | 0.98 | 3.1E-01 | 4.2E-05 | |  |
| rs17590608 | 3.24 | 0.82 | 9.0E-05 | C | 0.41 | | 1.02 | | 0.78 | | 3.4E-01 | 1.53 | 1.22 | 0.78 | 1.1E-01 | 4.3E-05 | |  |
| rs11252394 | 6.01 | 1.42 | 2.3E-05 | A | 0.21 | | 1.40 | | 0.96 | | 4.4E-01 | -0.35 | 1.97 | 0.98 | 5.7E-01 | 4.3E-05 | |  |
| rs10781126 | 3.33 | 0.78 | 2.3E-05 | A | -0.27 | | 0.97 | | 0.82 | | 6.1E-01 | 0.19 | 1.15 | 0.91 | 4.3E-01 | 4.4E-05 | |  |
| rs4775229 | 4.64 | 1.16 | 6.9E-05 | A | -1.28 | | 1.40 | | 0.95 | | 1.8E-01 | -0.70 | 1.78 | 0.98 | 3.5E-01 | 4.5E-05 | |  |
| rs11564299 | 3.44 | 0.90 | 1.4E-04 | A | -3.26 | | 1.15 | | 0.86 | | 2.5E-03 | 0.56 | 1.31 | 0.83 | 6.7E-01 | 4.8E-05 | |  |
| rs1348879 | 3.96 | 0.98 | 5.2E-05 | A | -0.48 | | 1.12 | | 0.96 | | 6.7E-01 | 1.50 | 1.35 | 1.00 | 1.3E-01 | 4.9E-05 | |  |
| rs7685518 | -3.31 | 0.86 | 1.3E-04 | A | 1.25 | | 0.98 | | 0.96 | | 1.0E-01 | 0.89 | 1.13 | 0.99 | 2.2E-01 | 4.9E-05 | |  |
| rs7598349 | 2.58 | 0.73 | 4.3E-04 | A | -2.04 | | 0.86 | | 0.87 | | 9.4E-03 | -1.32 | 0.94 | 0.95 | 8.2E-02 | 5.1E-05 | |  |
| rs2242930 | 3.29 | 0.80 | 4.4E-05 | A | -0.01 | | 0.94 | | 0.96 | | 5.1E-01 | 0.47 | 0.95 | 0.99 | 3.1E-01 | 5.2E-05 | |  |
| rs6661901 | 4.08 | 1.02 | 6.3E-05 | A | -0.65 | | 1.29 | | 0.95 | | 3.1E-01 | -0.60 | 1.36 | 0.99 | 3.3E-01 | 5.3E-05 | |  |
| rs7667104 | -3.29 | 0.86 | 1.4E-04 | A | 1.19 | | 0.98 | | 0.96 | | 1.1E-01 | 0.86 | 1.13 | 0.99 | 2.2E-01 | 5.8E-05 | |  |
| rs7644264 | -4.20 | 1.07 | 9.1E-05 | C | 0.46 | | 1.19 | | 0.93 | | 3.5E-01 | 1.15 | 1.35 | 0.96 | 2.0E-01 | 6.0E-05 | |  |
| rs7677190 | -3.26 | 0.87 | 1.7E-04 | G | -1.25 | | 0.98 | | 0.96 | | 1.0E-01 | -0.88 | 1.13 | 0.99 | 2.2E-01 | 6.6E-05 | |  |
| rs7730346 | 4.64 | 1.22 | 1.5E-04 | G | -4.50 | | 2.27 | | 0.62 | | 2.4E-02 | 0.15 | 1.56 | 0.97 | 5.4E-01 | 7.0E-05 | |  |
| rs4958980 | 4.64 | 1.22 | 1.5E-04 | A | 4.52 | | 2.40 | | 0.56 | | 3.1E-02 | -0.15 | 1.59 | 0.95 | 5.4E-01 | 7.5E-05 | |  |
| rs159320 | 2.51 | 0.72 | 5.2E-04 | A | -2.40 | | 0.80 | | 0.96 | | 1.5E-03 | -0.59 | 0.96 | 1.00 | 2.7E-01 | 7.7E-05 | |  |
| rs6414806 | -2.97 | 0.72 | 3.7E-05 | C | -0.19 | | 0.88 | | 0.89 | | 5.9E-01 | -0.11 | 0.97 | 0.93 | 5.5E-01 | 8.1E-05 | |  |
| rs565659 | -3.10 | 0.84 | 2.1E-04 | A | 1.98 | | 0.93 | | 0.97 | | 1.8E-02 | 0.08 | 1.07 | 0.99 | 4.7E-01 | 8.1E-05 | |  |
| rs11083252 | 3.34 | 0.90 | 2.1E-04 | A | 3.12 | | 1.04 | | 0.96 | | 1.5E-03 | -0.85 | 1.20 | 0.90 | 7.6E-01 | 8.2E-05 | |  |
| rs9838009 | 3.91 | 1.01 | 1.2E-04 | G | -1.79 | | 1.15 | | 0.96 | | 6.0E-02 | 0.37 | 1.39 | 0.89 | 6.0E-01 | 8.4E-05 | |  |
| rs7754623 | 3.93 | 1.05 | 1.8E-04 | A | -1.03 | | 1.32 | | 0.93 | | 7.8E-01 | 3.13 | 1.38 | 0.97 | 1.2E-02 | 8.8E-05 | |  |
| rs10097529 | -3.92 | 1.05 | 1.8E-04 | A | -0.96 | | 1.38 | | 0.98 | | 2.4E-01 | -1.35 | 1.39 | 1.00 | 1.7E-01 | 8.8E-05 | |  |
| rs1586150 | -3.20 | 0.87 | 2.4E-04 | C | 1.24 | | 0.99 | | 0.96 | | 1.1E-01 | 0.92 | 1.12 | 0.99 | 2.1E-01 | 9.1E-05 | |  |
| rs4146626 | 3.13 | 0.75 | 3.3E-05 | A | 0.46 | | 0.86 | | 0.95 | | 7.0E-01 | 0.17 | 0.95 | 0.98 | 5.7E-01 | 9.6E-05 | |  |
| rs2811686 | 3.27 | 0.86 | 1.5E-04 | G | -0.01 | | 0.97 | | 0.95 | | 5.0E-01 | 1.39 | 1.15 | 0.95 | 1.1E-01 | 9.8E-05 | |  |
| rs2826840 | -4.25 | 1.04 | 4.8E-05 | A | -1.11 | | 1.25 | | 0.85 | | 8.1E-01 | 0.76 | 1.48 | 0.89 | 3.0E-01 | 1.0E-04 | |  |
