# Supplementary material for: Genome-Wide Association Analysis in Asthma Subjects Identifies SPATS2L as a Novel Bronchodilator Response Gene
Source: PLoS Genet. 2012 Jul 5;8(7):e1002824. doi: 10.1371/journal.pgen.1002824 (PMC3390407; doi:10.1371/journal.pgen.1002824)
Supplement: Table S3 — Primary GWAS 1000GP Imputed SNP details for SNPs with P<1E-05. MAF = Minor Allele Frequency. Rsq = MACH R-squared value for imputed SNP. (DOCX) [file pgen.1002824.s010.docx]

|  |  |  |  |  | CAMP/LOCCS/LODO/Sepracor | | | CARE | | | ACRN | | |
| --- | --- | --- | --- | --- | --- | --- | --- | --- | --- | --- | --- | --- | --- |
| SNP | CHR | BP | Reference Allele | Alternate Allele | Reference Allele Frequency | MAF | Rsq | Reference Allele Frequency | MAF | Rsq | Reference Allele Frequency | MAF | Rsq |
| chr8:18369015 | 8 | 18369015 | G | A | 0.93 | 0.07 | 0.59 | 0.92 | 0.08 | 0.67 | 0.91 | 0.09 | 0.75 |
| rs298566 | 5 | 17351004 | C | T | 0.34 | 0.34 | 0.91 | 0.31 | 0.31 | 0.96 | 0.32 | 0.32 | 0.97 |
| rs2652653 | 5 | 17349431 | A | G | 0.34 | 0.34 | 0.91 | 0.31 | 0.31 | 0.96 | 0.32 | 0.32 | 0.97 |
| chr8:107404520 | 8 | 107404520 | A | G | 0.76 | 0.24 | 0.98 | 0.77 | 0.23 | 0.96 | 0.77 | 0.23 | 0.99 |
| rs298568 | 5 | 17346761 | A | C | 0.34 | 0.34 | 0.91 | 0.31 | 0.31 | 0.95 | 0.32 | 0.32 | 0.97 |
| rs9297380 | 8 | 107403004 | C | T | 0.76 | 0.24 | 1.00 | 0.76 | 0.24 | 0.96 | 0.76 | 0.24 | 1.00 |
| rs9283944 | 8 | 107403285 | C | T | 0.76 | 0.24 | 1.00 | 0.76 | 0.24 | 0.96 | 0.76 | 0.24 | 1.00 |
| rs9297381 | 8 | 107403507 | G | A | 0.76 | 0.24 | 1.00 | 0.76 | 0.24 | 0.96 | 0.76 | 0.24 | 1.00 |
| rs2345581 | 8 | 107403862 | A | G | 0.76 | 0.24 | 1.00 | 0.76 | 0.24 | 0.96 | 0.76 | 0.24 | 1.00 |
| rs10105383 | 8 | 107404746 | A | T | 0.76 | 0.24 | 1.00 | 0.76 | 0.24 | 0.96 | 0.76 | 0.24 | 1.00 |
| rs7874116 | 9 | 75003130 | C | A | 0.76 | 0.24 | 0.96 | 0.76 | 0.24 | 0.90 | 0.77 | 0.23 | 0.94 |
| rs9774587 | 8 | 107406064 | C | A | 0.77 | 0.23 | 0.97 | 0.78 | 0.22 | 0.93 | 0.80 | 0.20 | 0.97 |
| rs4452682 | 6 | 3360301 | A | G | 0.40 | 0.40 | 0.99 | 0.43 | 0.43 | 0.92 | 0.44 | 0.44 | 0.90 |
| rs7715410 | 5 | 66843666 | C | A | 0.62 | 0.38 | 0.98 | 0.65 | 0.35 | 0.80 | 0.64 | 0.36 | 0.78 |
| rs295137 | 2 | 200858285 | T | C | 0.41 | 0.41 | 1.00 | 0.41 | 0.41 | 0.89 | 0.41 | 0.41 | 0.97 |
| rs295142 | 2 | 200919961 | G | A | 0.41 | 0.41 | 0.98 | 0.41 | 0.41 | 0.95 | 0.39 | 0.39 | 1.00 |
| rs1900706 | 2 | 200922316 | C | A | 0.41 | 0.41 | 0.98 | 0.40 | 0.40 | 0.97 | 0.39 | 0.39 | 1.00 |
| rs12824038 | 12 | 72748637 | G | A | 0.89 | 0.11 | 0.92 | 0.89 | 0.11 | 0.89 | 0.88 | 0.12 | 0.92 |
| rs11179902 | 12 | 72751799 | C | A | 0.89 | 0.11 | 0.92 | 0.89 | 0.11 | 0.89 | 0.88 | 0.12 | 0.92 |
| rs16874388 | 8 | 107406116 | T | C | 0.76 | 0.24 | 1.00 | 0.77 | 0.23 | 0.94 | 0.78 | 0.22 | 0.99 |
| chr3:25080552 | 3 | 25080552 | C | T | 0.85 | 0.15 | 0.99 | 0.85 | 0.15 | 0.96 | 0.86 | 0.14 | 0.98 |
| rs295139 | 2 | 200868233 | A | T | 0.42 | 0.42 | 0.99 | 0.42 | 0.42 | 0.86 | 0.42 | 0.42 | 0.97 |
| rs10283328 | 8 | 107423034 | G | A | 0.75 | 0.25 | 0.96 | 0.76 | 0.24 | 0.94 | 0.75 | 0.25 | 0.95 |
| rs6992673 | 8 | 107422489 | G | A | 0.75 | 0.25 | 0.96 | 0.76 | 0.24 | 0.94 | 0.76 | 0.24 | 0.95 |
| rs10092617 | 8 | 107408317 | A | G | 0.76 | 0.24 | 1.00 | 0.77 | 0.23 | 0.94 | 0.78 | 0.22 | 0.99 |
| rs4328902 | 4 | 25348267 | T | C | 0.27 | 0.27 | 0.99 | 0.27 | 0.27 | 0.65 | 0.26 | 0.26 | 0.66 |
| rs4382051 | 4 | 127221258 | A | G | 0.79 | 0.21 | 0.96 | 0.78 | 0.22 | 0.92 | 0.79 | 0.21 | 0.93 |
| rs10940113 | 5 | 66850598 | T | C | 0.63 | 0.37 | 1.00 | 0.66 | 0.34 | 0.91 | 0.65 | 0.35 | 0.90 |
| chr2:34482353 | 2 | 34482353 | C | T | 0.85 | 0.15 | 0.88 | 0.86 | 0.14 | 0.68 | 0.86 | 0.14 | 0.87 |
| rs12509991 | 4 | 127216320 | C | T | 0.80 | 0.20 | 0.99 | 0.80 | 0.20 | 0.94 | 0.80 | 0.20 | 0.95 |
| rs11179933 | 12 | 72815097 | C | T | 0.85 | 0.15 | 0.96 | 0.84 | 0.16 | 0.93 | 0.83 | 0.17 | 0.96 |
| chr18:24507294 | 18 | 24507294 | C | A | 0.91 | 0.09 | 0.59 | 0.91 | 0.09 | 0.53 | 0.91 | 0.09 | 0.51 |
