# Supplementary material for: Genome-Wide Association Analysis in Asthma Subjects Identifies SPATS2L as a Novel Bronchodilator Response Gene
Source: PLoS Genet. 2012 Jul 5;8(7):e1002824. doi: 10.1371/journal.pgen.1002824 (PMC3390407; doi:10.1371/journal.pgen.1002824)
Supplement: Table S4 — Primary GWAS 1000GP Imputed SNP P-values and Beta coefficients for SNPs with P<1E-05. CARE and ACRN P-values are 1-sided based on the direction in CAMP/LOCCS/LODO/Sepracor. SE = Standard Error for corresponding Beta coefficient. Combined P-values were obtained using the Liptak method, with weights proportional to population size. (DOCX) [file pgen.1002824.s011.docx]

|  |  | CAMP/LOCCS/LODO/Sepracor | | | CARE | | | ACRN | | |  |  |  |
| --- | --- | --- | --- | --- | --- | --- | --- | --- | --- | --- | --- | --- | --- |
| SNP | Reference Allele | BETA | SE | P-value | BETA | SE | P-value | BETA | SE | P-value | Combined P-value | Primary GWAS Combined P-value | Primary GWAS Rank |
| chr8:18369015 | G | -8.31 | 1.79 | 4.0E-06 | -1.68 | 2.00 | 2.0E-01 | -3.16 | 1.91 | 5.0E-02 | 8.9E-07 | - | - |
| rs298566 | C | 3.97 | 0.80 | 8.4E-07 | -0.02 | 0.88 | 5.1E-01 | -0.13 | 0.99 | 5.5E-01 | 2.1E-06 | - | - |
| rs2652653 | A | 3.97 | 0.80 | 8.6E-07 | -0.02 | 0.88 | 5.1E-01 | -0.15 | 0.99 | 5.6E-01 | 2.2E-06 | - | - |
| chr8:107404520 | A | 3.80 | 0.85 | 9.4E-06 | 0.61 | 1.03 | 2.8E-01 | 2.08 | 1.14 | 3.5E-02 | 2.2E-06 | - | - |
| rs298568 | A | 3.97 | 0.80 | 9.0E-07 | -0.02 | 0.88 | 5.1E-01 | -0.17 | 0.99 | 5.7E-01 | 2.4E-06 | - | - |
| rs9297380 | C | 3.68 | 0.84 | 1.3E-05 | 0.79 | 1.02 | 2.2E-01 | 2.09 | 1.12 | 3.2E-02 | 2.7E-06 | - | - |
| rs9283944 | C | 3.68 | 0.84 | 1.4E-05 | 0.79 | 1.02 | 2.2E-01 | 2.09 | 1.12 | 3.2E-02 | 2.8E-06 | - | - |
| rs9297381 | G | 3.68 | 0.84 | 1.4E-05 | 0.79 | 1.02 | 2.2E-01 | 2.09 | 1.12 | 3.2E-02 | 2.8E-06 | - | - |
| rs2345581 | A | 3.68 | 0.84 | 1.4E-05 | 0.79 | 1.02 | 2.2E-01 | 2.09 | 1.12 | 3.2E-02 | 2.8E-06 | - | - |
| rs10105383 | A | 3.67 | 0.84 | 1.4E-05 | 0.79 | 1.02 | 2.2E-01 | 2.09 | 1.12 | 3.2E-02 | 2.9E-06 | - | - |
| rs7874116 | C | -3.77 | 0.86 | 1.3E-05 | 0.68 | 1.04 | 7.4E-01 | -3.23 | 1.18 | 3.2E-03 | 3.7E-06 | - | - |
| rs9774587 | C | 3.86 | 0.86 | 8.1E-06 | 0.40 | 1.12 | 3.6E-01 | 1.58 | 1.22 | 9.8E-02 | 3.8E-06 | - | - |
| rs4452682 | A | -2.99 | 0.74 | 5.6E-05 | -1.71 | 0.90 | 2.9E-02 | -2.05 | 0.97 | 1.8E-02 | 4.1E-06 | 3.2E-06 | 1 |
| rs7715410 | C | 3.75 | 0.75 | 6.6E-07 | -0.36 | 0.92 | 6.5E-01 | -0.81 | 1.03 | 7.8E-01 | 4.1E-06 | - | - |
| rs295137 | T | 2.94 | 0.72 | 5.1E-05 | 2.50 | 0.84 | 1.6E-03 | 0.92 | 0.97 | 1.7E-01 | 4.6E-06 | 3.4E-06 | 2 |
| rs295142 | G | 3.11 | 0.74 | 2.8E-05 | 2.53 | 0.81 | 9.9E-04 | 0.07 | 0.95 | 4.7E-01 | 4.7E-06 | - | - |
| rs1900706 | C | 3.10 | 0.74 | 2.9E-05 | 2.54 | 0.80 | 9.2E-04 | 0.07 | 0.95 | 4.7E-01 | 4.9E-06 | - | - |
| rs12824038 | G | -5.42 | 1.21 | 8.4E-06 | -1.57 | 1.33 | 1.2E-01 | -0.32 | 1.48 | 4.1E-01 | 5.5E-06 | - | - |
| rs11179902 | C | -5.42 | 1.21 | 8.4E-06 | -1.57 | 1.33 | 1.2E-01 | -0.32 | 1.48 | 4.1E-01 | 5.5E-06 | - | - |
| rs16874388 | T | 3.66 | 0.84 | 1.5E-05 | 0.52 | 1.06 | 3.1E-01 | 1.69 | 1.16 | 7.4E-02 | 5.5E-06 | - | - |
| chr3:25080552 | C | -4.44 | 1.02 | 1.4E-05 | -0.33 | 1.10 | 3.8E-01 | -1.72 | 1.30 | 9.4E-02 | 6.7E-06 | - | - |
| rs295139 | A | 2.96 | 0.73 | 4.9E-05 | 2.46 | 0.85 | 2.2E-03 | 0.56 | 0.98 | 2.9E-01 | 6.8E-06 | - | - |
| rs10283328 | G | 3.52 | 0.85 | 3.7E-05 | 1.06 | 1.03 | 1.5E-01 | 2.02 | 1.13 | 3.7E-02 | 6.9E-06 | - | - |
| rs6992673 | G | 3.53 | 0.85 | 3.7E-05 | 1.05 | 1.03 | 1.5E-01 | 2.02 | 1.14 | 3.9E-02 | 7.0E-06 | - | - |
| rs10092617 | A | 3.61 | 0.84 | 2.0E-05 | 0.53 | 1.06 | 3.1E-01 | 1.69 | 1.16 | 7.4E-02 | 7.3E-06 | - | - |
| rs4328902 | T | -3.47 | 0.82 | 2.4E-05 | -0.40 | 1.24 | 3.7E-01 | -2.26 | 1.29 | 4.1E-02 | 7.8E-06 | 6.1E-06 | 5 |
| rs4382051 | A | 4.02 | 0.91 | 9.8E-06 | 0.47 | 1.04 | 3.2E-01 | 0.66 | 1.15 | 2.8E-01 | 8.0E-06 | - | - |
| rs10940113 | T | 3.61 | 0.75 | 1.5E-06 | -0.43 | 0.86 | 6.9E-01 | -0.70 | 0.97 | 7.6E-01 | 8.9E-06 | 6.1E-06 | 4 |
| chr2:34482353 | C | 4.67 | 1.06 | 1.1E-05 | 1.49 | 1.48 | 1.6E-01 | 0.08 | 1.53 | 4.8E-01 | 9.2E-06 | - | - |
| rs12509991 | C | 4.14 | 0.93 | 9.7E-06 | 0.28 | 1.07 | 4.0E-01 | 0.57 | 1.14 | 3.1E-01 | 9.7E-06 | - | - |
| rs11179933 | C | -4.39 | 1.03 | 2.3E-05 | -1.21 | 1.15 | 1.5E-01 | -0.98 | 1.20 | 2.1E-01 | 9.8E-06 | 2.5E-05 | 19 |
| chr18:24507294 | C | 6.77 | 1.64 | 3.7E-05 | 0.81 | 1.87 | 3.3E-01 | 4.51 | 2.38 | 3.0E-02 | 9.8E-06 | - | - |
