# Supplementary material for: Genome-Wide Association Analysis in Asthma Subjects Identifies SPATS2L as a Novel Bronchodilator Response Gene
Source: PLoS Genet. 2012 Jul 5;8(7):e1002824. doi: 10.1371/journal.pgen.1002824 (PMC3390407; doi:10.1371/journal.pgen.1002824)
Supplement: Table S5 — SARP Replication Results for SNPs with Primary GWAS P<1E-04. P-values are 1-sided based on the direction in CAMP/LOCCS/LODO/Sepracor. MAF = minor allele frequency. (DOCX) [file pgen.1002824.s012.docx]

| SNP | Minor Allele | MAF | Beta | P-value |
| --- | --- | --- | --- | --- |
| rs4452682 | A | 0.50 | -0.18 | 0.43 |
| rs295137 | T | 0.41 | 1.69 | 0.044 |
| rs295114 | T | 0.43 | 1.68 | 0.043 |
| rs10940113 | C | 0.37 | -0.93 | 0.16 |
| rs4328902 | T | 0.34 | 1.37 | 0.91 |
| rs2178806 | T | 0.18 | 0.92 | 0.25 |
| rs12306576 | G | 0.12 | -0.62 | 0.66 |
| rs10518485 | T | 0.20 | -1.42 | 0.10 |
| rs4976079 | T | 0.48 | -1.28 | 0.081 |
| rs12682157 | G | 0.23 | -1.38 | 0.12 |
| rs4581121 | C | 0.22 | -0.07 | 0.53 |
| rs7305291 | T | 0.12 | -0.50 | 0.63 |
| rs7958129 | T | 0.21 | 0.22 | 0.58 |
| rs17535206 | G | 0.10 | -1.44 | 0.75 |
| rs11179922 | C | 0.08 | -0.76 | 0.68 |
| rs6061043 | C | 0.48 | 0.30 | 0.38 |
| rs7013315 | G | 0.23 | -1.39 | 0.11 |
| rs10211073 | G | 0.47 | -0.46 | 0.31 |
| rs11179933 | T | 0.15 | -0.93 | 0.76 |
| rs1320269 | G | 0.19 | -1.54 | 0.09 |
| rs1889318 | C | 0.41 | -0.79 | 0.80 |
| rs4976096 | T | 0.26 | -1.26 | 0.11 |
| rs2367910 | A | 0.10 | -1.04 | 0.77 |
| rs6534528 | G | 0.20 | -1.73 | 0.06 |
| rs17531061 | C | 0.28 | 2.43 | 0.015 |
| rs12496948 | A | 0.23 | -1.60 | 0.067 |
| rs1857922 | T | 0.36 | -0.28 | 0.39 |
| rs1334086 | A | 0.44 | -1.53 | 0.95 |
| rs17010666 | C | 0.21 | -1.25 | 0.13 |
| rs17590608 | C | 0.29 | 0.40 | 0.36 |
| rs11252394 | - | - | - | - |
| rs10781126 | A | 0.25 | -0.02 | 0.51 |
| rs4775229 | C | 0.11 | 0.59 | 0.33 |
| rs11564299 | G | 0.25 | 0.80 | 0.24 |
| rs1348879 | A | 0.20 | 1.78 | 0.073 |
| rs7685518 | G | 0.22 | -0.05 | 0.48 |
| rs7598349 | G | 0.44 | 0.79 | 0.21 |
| rs2242930 | A | 0.26 | 0.97 | 0.18 |
| rs6661901 | G | 0.16 | 0.22 | 0.43 |
| rs7667104 | G | 0.23 | -0.03 | 0.49 |
| rs7644264 | T | 0.14 | -0.29 | 0.41 |
| rs7677190 | G | 0.21 | 0.05 | 0.52 |
| rs7730346 | T | 0.07 | -1.95 | 0.88 |
| rs4958980 | - | - | - | - |
| rs159320 | G | 0.43 | 0.99 | 0.15 |
| rs6414806 | T | 0.44 | -1.15 | 0.10 |
| rs565659 | C | 0.24 | 0.19 | 0.57 |
| rs11083252 | A | 0.27 | 0.68 | 0.27 |
| rs9838009 | T | 0.13 | -1.28 | 0.85 |
| rs7754623 | A | 0.14 | 1.68 | 0.13 |
| rs10097529 | A | 0.09 | 0.50 | 0.63 |
| rs1586150 | T | 0.21 | 0.11 | 0.54 |
| rs4146626 | G | 0.38 | 1.03 | 0.14 |
| rs2811686 | G | 0.31 | 0.03 | 0.49 |
| rs2826840 | G | 0.16 | 1.22 | 0.83 |
