# Supplementary material for: Genome-Wide Association Analysis in Asthma Subjects Identifies SPATS2L as a Novel Bronchodilator Response Gene
Source: PLoS Genet. 2012 Jul 5;8(7):e1002824. doi: 10.1371/journal.pgen.1002824 (PMC3390407; doi:10.1371/journal.pgen.1002824)
Supplement: Table S6 — Unadjusted P-values and log fold-change quantifying differential expression of SPATS2L and SPATS2 probes for GEO dataset GSE13168, in which human ASM cell lines expressing a PKA inhibitor vs. a GFP control were compared at baseline and when stimulated with IL1b, EGF, or both. (DOCX) [file pgen.1002824.s013.docx]

|  | Basal | | EGF | | IL1b | | Both | |
| --- | --- | --- | --- | --- | --- | --- | --- | --- |
| Probe | P-value | LogFC | P-value | LogFC | P-value | LogFC | P-value | LogFC |
| *SPATS2* (218324_s_at) | 0.028 | 0.62 | 4.2E-04 | 1.08 | 0.014 | 0.81 | 1.1E-03 | 0.99 |
| *SPATS2L* (215617_at) | 0.47 | 0.20 | 0.093 | 0.48 | 0.096 | 0.55 | 7.1E-03 | 0.81 |
| *SPATS2L* (222154_s_at) | 0.28 | -0.22 | 0.27 | 0.22 | 0.88 | 0.03 | 0.12 | 0.31 |
