# Supplementary material for: Genome-Wide Association Analysis in Asthma Subjects Identifies SPATS2L as a Novel Bronchodilator Response Gene
Source: PLoS Genet. 2012 Jul 5;8(7):e1002824. doi: 10.1371/journal.pgen.1002824 (PMC3390407; doi:10.1371/journal.pgen.1002824)
Supplement: Table S7 — Primary GWAS 1000GP Imputed SNP details for SNPs that have nominally significant p-values (<0.05) in, or within 50,000 KB of, genes (i.e. ADRB2, ADCY9, CRHR2, ARG1) previously identified as being associated with BDR. There were no such SNPs near ADRB2. MAF = Minor Allele Frequency. Rsq = MACH R-squared value for imputed SNP. (DOCX) [file pgen.1002824.s014.docx]

|  |  |  |  |  |  | CAMP/LOCCS/LODO/Sepracor | | | CARE | | | ACRN | | |
| --- | --- | --- | --- | --- | --- | --- | --- | --- | --- | --- | --- | --- | --- | --- |
| SNP | CHR | BP | Gene | Reference Allele | Alternate Allele | Reference Allele Frequency | MAF | Rsq | Reference Allele Frequency | MAF | Rsq | Reference Allele Frequency | MAF | Rsq |
| rs2531993 | 16 | 3954246 | *ADCY9* | G | A | 0.85 | 0.15 | 0.95 | 0.81 | 0.19 | 0.69 | 0.85 | 0.15 | 0.53 |
| rs710893 | 16 | 3954965 | *ADCY9* | G | A | 0.85 | 0.15 | 0.98 | 0.83 | 0.17 | 0.74 | 0.85 | 0.15 | 0.56 |
| rs2230742 | 16 | 3956677 | *ADCY9* | G | A | 0.83 | 0.17 | 0.88 | 0.81 | 0.19 | 0.71 | 0.84 | 0.17 | 0.51 |
| rs2531992 | 16 | 3961735 | *ADCY9* | G | A | 0.85 | 0.15 | 0.99 | 0.83 | 0.17 | 0.75 | 0.85 | 0.15 | 0.57 |
| chr16:3962639 | 16 | 3962639 | *ADCY9* | T | C | 0.84 | 0.16 | 0.95 | 0.83 | 0.17 | 0.82 | 0.86 | 0.14 | 0.70 |
| rs9935335 | 16 | 3963029 | *ADCY9* | T | A | 0.81 | 0.19 | 0.97 | 0.81 | 0.19 | 0.78 | 0.84 | 0.16 | 0.67 |
| rs2531991 | 16 | 3963554 | *ADCY9* | A | G | 0.76 | 0.24 | 0.98 | 0.76 | 0.24 | 0.72 | 0.80 | 0.20 | 0.51 |
| rs2238436 | 16 | 3965386 | *ADCY9* | G | T | 0.76 | 0.24 | 0.98 | 0.76 | 0.24 | 0.72 | 0.80 | 0.20 | 0.51 |
| rs2072346 | 16 | 3967424 | *ADCY9* | T | C | 0.82 | 0.18 | 0.99 | 0.82 | 0.18 | 0.82 | 0.85 | 0.15 | 0.72 |
| rs2240735 | 16 | 3967606 | *ADCY9* | T | C | 0.76 | 0.24 | 0.99 | 0.76 | 0.24 | 0.73 | 0.80 | 0.20 | 0.52 |
| rs2601775 | 16 | 3968061 | *ADCY9* | G | A | 0.78 | 0.22 | 0.98 | 0.79 | 0.22 | 0.77 | 0.81 | 0.19 | 0.59 |
| rs2531989 | 16 | 3968146 | *ADCY9* | A | G | 0.84 | 0.16 | 0.98 | 0.84 | 0.16 | 0.92 | 0.87 | 0.14 | 0.84 |
| rs2531988 | 16 | 3968418 | *ADCY9* | G | T | 0.84 | 0.16 | 0.94 | 0.82 | 0.18 | 0.83 | 0.85 | 0.15 | 0.75 |
| rs12922685 | 16 | 3968622 | *ADCY9* | G | C | 0.84 | 0.16 | 0.99 | 0.84 | 0.16 | 0.92 | 0.86 | 0.14 | 0.85 |
| rs2531986 | 16 | 3969578 | *ADCY9* | C | T | 0.82 | 0.18 | 0.97 | 0.83 | 0.17 | 0.87 | 0.86 | 0.14 | 0.81 |
| rs2238439 | 16 | 3971349 | *ADCY9* | G | A | 0.78 | 0.22 | 0.99 | 0.78 | 0.22 | 0.78 | 0.81 | 0.19 | 0.63 |
| rs2041248 | 16 | 3972490 | *ADCY9* | G | T | 0.84 | 0.16 | 0.98 | 0.83 | 0.17 | 0.89 | 0.86 | 0.14 | 0.87 |
| rs2041247 | 16 | 3972582 | *ADCY9* | C | T | 0.84 | 0.16 | 0.99 | 0.84 | 0.16 | 0.94 | 0.86 | 0.14 | 0.93 |
| rs933392 | 16 | 3972717 | *ADCY9* | G | T | 0.85 | 0.15 | 1.00 | 0.84 | 0.16 | 0.94 | 0.86 | 0.14 | 0.93 |
| rs2531983 | 16 | 3973013 | *ADCY9* | C | A | 0.83 | 0.17 | 0.95 | 0.83 | 0.17 | 0.84 | 0.85 | 0.15 | 0.87 |
| rs12325363 | 16 | 4055029 | *ADCY9* | G | A | 0.86 | 0.14 | 0.97 | 0.85 | 0.15 | 0.93 | 0.84 | 0.16 | 0.97 |
| rs2159296 | 16 | 4063324 | *ADCY9* | G | A | 0.75 | 0.25 | 1.00 | 0.75 | 0.25 | 0.97 | 0.73 | 0.27 | 0.99 |
| rs2532022 | 16 | 4063421 | *ADCY9* | G | A | 0.75 | 0.25 | 1.00 | 0.75 | 0.25 | 0.97 | 0.73 | 0.27 | 0.99 |
| rs2532020 | 16 | 4064108 | *ADCY9* | G | A | 0.75 | 0.25 | 1.00 | 0.75 | 0.25 | 0.97 | 0.73 | 0.27 | 0.99 |
| rs2532019 | 16 | 4064301 | *ADCY9* | T | G | 0.75 | 0.25 | 1.00 | 0.75 | 0.25 | 0.97 | 0.73 | 0.27 | 0.99 |
| rs2601813 | 16 | 4064338 | *ADCY9* | C | G | 0.75 | 0.25 | 1.00 | 0.75 | 0.25 | 0.97 | 0.73 | 0.27 | 0.99 |
| rs6500578 | 16 | 4064591 | *ADCY9* | T | C | 0.75 | 0.25 | 1.00 | 0.75 | 0.25 | 0.97 | 0.73 | 0.27 | 0.99 |
| rs2781640 | 6 | 131886060 | *ARG1* | G | A | 0.78 | 0.22 | 0.95 | 0.79 | 0.21 | 0.87 | 0.81 | 0.19 | 0.89 |
| rs2749929 | 6 | 131901507 | *ARG1* | T | C | 0.79 | 0.22 | 0.98 | 0.78 | 0.22 | 0.97 | 0.79 | 0.21 | 0.99 |
| rs2781646 | 6 | 131909912 | *ARG1* | A | G | 0.78 | 0.22 | 0.98 | 0.78 | 0.22 | 0.97 | 0.79 | 0.21 | 0.99 |
| rs2608909 | 6 | 131909963 | *ARG1* | A | G | 0.78 | 0.22 | 0.99 | 0.78 | 0.22 | 0.97 | 0.79 | 0.21 | 0.99 |
| rs2608910 | 6 | 131910095 | *ARG1* | T | G | 0.78 | 0.22 | 0.99 | 0.78 | 0.22 | 0.97 | 0.79 | 0.21 | 0.99 |
| rs2781647 | 6 | 131910565 | *ARG1* | G | A | 0.78 | 0.22 | 1.00 | 0.78 | 0.22 | 0.97 | 0.79 | 0.21 | 0.99 |
| rs2608912 | 6 | 131911476 | *ARG1* | T | C | 0.79 | 0.21 | 1.00 | 0.78 | 0.22 | 0.97 | 0.79 | 0.21 | 1.00 |
| rs2608913 | 6 | 131911954 | *ARG1* | T | C | 0.79 | 0.21 | 1.00 | 0.78 | 0.22 | 0.97 | 0.79 | 0.21 | 0.99 |
| rs2608914 | 6 | 131912304 | *ARG1* | T | A | 0.78 | 0.22 | 1.00 | 0.78 | 0.22 | 0.97 | 0.79 | 0.21 | 0.99 |
| rs2608915 | 6 | 131913007 | *ARG1* | A | G | 0.79 | 0.21 | 1.00 | 0.78 | 0.22 | 0.97 | 0.79 | 0.21 | 0.99 |
| rs2608916 | 6 | 131913298 | *ARG1* | T | G | 0.79 | 0.21 | 1.00 | 0.78 | 0.22 | 0.97 | 0.79 | 0.21 | 0.99 |
| rs2608917 | 6 | 131913649 | *ARG1* | A | G | 0.79 | 0.21 | 1.00 | 0.78 | 0.22 | 0.97 | 0.79 | 0.21 | 0.99 |
| rs2608918 | 6 | 131914585 | *ARG1* | C | T | 0.78 | 0.22 | 1.00 | 0.78 | 0.22 | 0.97 | 0.79 | 0.21 | 0.99 |
| rs2608919 | 6 | 131915028 | *ARG1* | A | T | 0.78 | 0.22 | 0.97 | 0.78 | 0.22 | 0.97 | 0.78 | 0.22 | 0.99 |
| rs2608920 | 6 | 131915396 | *ARG1* | T | C | 0.78 | 0.22 | 0.97 | 0.78 | 0.22 | 0.97 | 0.78 | 0.22 | 0.99 |
| rs2608921 | 6 | 131915428 | *ARG1* | C | T | 0.78 | 0.22 | 0.97 | 0.78 | 0.22 | 0.97 | 0.78 | 0.22 | 0.99 |
| rs2446213 | 6 | 131915724 | *ARG1* | T | C | 0.78 | 0.22 | 0.97 | 0.78 | 0.22 | 0.97 | 0.78 | 0.22 | 1.00 |
| rs2608983 | 6 | 131917337 | *ARG1* | T | C | 0.78 | 0.22 | 0.97 | 0.78 | 0.22 | 0.97 | 0.78 | 0.22 | 1.00 |
| rs2749932 | 6 | 131917855 | *ARG1* | C | T | 0.78 | 0.22 | 0.97 | 0.78 | 0.22 | 0.97 | 0.78 | 0.22 | 0.99 |
| rs2608982 | 6 | 131917893 | *ARG1* | G | T | 0.82 | 0.18 | 0.79 | 0.80 | 0.20 | 0.85 | 0.80 | 0.20 | 0.87 |
| rs2608981 | 6 | 131917990 | *ARG1* | T | G | 0.78 | 0.22 | 0.97 | 0.78 | 0.22 | 0.97 | 0.78 | 0.22 | 0.99 |
| rs28612296 | 6 | 131918282 | *ARG1* | G | A | 0.78 | 0.22 | 0.96 | 0.77 | 0.23 | 0.97 | 0.78 | 0.22 | 1.00 |
| rs2781649 | 6 | 131918330 | *ARG1* | A | G | 0.78 | 0.22 | 0.97 | 0.78 | 0.22 | 0.97 | 0.78 | 0.22 | 1.00 |
| rs2781650 | 6 | 131918414 | *ARG1* | A | G | 0.78 | 0.22 | 0.97 | 0.78 | 0.22 | 0.97 | 0.78 | 0.22 | 1.00 |
| rs2781651 | 6 | 131918636 | *ARG1* | A | G | 0.78 | 0.22 | 0.96 | 0.77 | 0.23 | 0.97 | 0.78 | 0.22 | 1.00 |
| rs2781652 | 6 | 131918809 | *ARG1* | A | G | 0.78 | 0.22 | 0.97 | 0.78 | 0.22 | 0.97 | 0.78 | 0.22 | 0.99 |
| rs2749933 | 6 | 131918926 | *ARG1* | C | T | 0.78 | 0.22 | 0.97 | 0.78 | 0.22 | 0.97 | 0.78 | 0.22 | 0.99 |
| rs2608977 | 6 | 131919354 | *ARG1* | T | C | 0.78 | 0.22 | 0.97 | 0.78 | 0.22 | 0.97 | 0.78 | 0.22 | 0.98 |
| rs2608976 | 6 | 131919868 | *ARG1* | T | C | 0.78 | 0.22 | 0.96 | 0.78 | 0.22 | 0.97 | 0.78 | 0.22 | 0.98 |
| rs2608975 | 6 | 131920974 | *ARG1* | G | A | 0.78 | 0.22 | 0.96 | 0.78 | 0.22 | 0.97 | 0.78 | 0.22 | 0.98 |
| rs2781654 | 6 | 131922733 | *ARG1* | C | T | 0.78 | 0.22 | 0.95 | 0.80 | 0.20 | 0.83 | 0.80 | 0.20 | 0.83 |
| rs9375818 | 6 | 131923771 | *ARG1* | G | A | 0.82 | 0.18 | 0.79 | 0.84 | 0.16 | 0.68 | 0.84 | 0.16 | 0.70 |
| rs6929820 | 6 | 131923773 | *ARG1* | T | C | 0.78 | 0.22 | 0.93 | 0.82 | 0.18 | 0.76 | 0.81 | 0.19 | 0.77 |
| rs2781656 | 6 | 131924264 | *ARG1* | C | T | 0.78 | 0.22 | 0.94 | 0.78 | 0.22 | 0.93 | 0.78 | 0.22 | 0.90 |
| rs2749935 | 6 | 131931574 | *ARG1* | A | T | 0.62 | 0.38 | 0.86 | 0.63 | 0.37 | 0.78 | 0.63 | 0.37 | 0.78 |
| rs2781659 | 6 | 131933513 | *ARG1* | A | G | 0.68 | 0.32 | 0.94 | 0.69 | 0.31 | 0.68 | 0.69 | 0.31 | 0.68 |
| rs2781660 | 6 | 131933532 | *ARG1* | T | C | 0.68 | 0.32 | 0.94 | 0.69 | 0.31 | 0.68 | 0.69 | 0.31 | 0.68 |
| rs2781661 | 6 | 131933565 | *ARG1* | G | T | 0.68 | 0.32 | 0.94 | 0.69 | 0.31 | 0.68 | 0.69 | 0.31 | 0.68 |
| rs2781662 | 6 | 131933761 | *ARG1* | A | G | 0.68 | 0.32 | 0.94 | 0.69 | 0.31 | 0.68 | 0.69 | 0.31 | 0.68 |
| rs2781663 | 6 | 131934040 | *ARG1* | T | A | 0.68 | 0.32 | 0.94 | 0.69 | 0.31 | 0.68 | 0.69 | 0.31 | 0.68 |
| rs2608898 | 6 | 131934119 | *ARG1* | C | G | 0.68 | 0.32 | 0.94 | 0.70 | 0.30 | 0.68 | 0.70 | 0.30 | 0.68 |
| rs2781664 | 6 | 131934669 | *ARG1* | T | C | 0.73 | 0.27 | 0.84 | 0.73 | 0.27 | 0.59 | 0.72 | 0.28 | 0.60 |
| rs2781665 | 6 | 131934940 | *ARG1* | A | T | 0.73 | 0.27 | 0.85 | 0.73 | 0.27 | 0.59 | 0.72 | 0.28 | 0.60 |
| rs2608897 | 6 | 131935156 | *ARG1* | C | T | 0.68 | 0.32 | 0.99 | 0.69 | 0.31 | 0.69 | 0.69 | 0.31 | 0.68 |
| rs2781666 | 6 | 131935252 | *ARG1* | G | T | 0.72 | 0.28 | 0.88 | 0.72 | 0.28 | 0.59 | 0.71 | 0.29 | 0.61 |
| rs2781667 | 6 | 131936837 | *ARG1* | C | T | 0.68 | 0.32 | 0.98 | 0.69 | 0.31 | 0.69 | 0.69 | 0.31 | 0.68 |
| rs1076291 | 7 | 30679120 | *CRHR2* | G | A | 0.65 | 0.35 | 0.99 | 0.63 | 0.37 | 0.95 | 0.63 | 0.38 | 0.98 |
| rs1076292 | 7 | 30679226 | *CRHR2* | G | C | 0.65 | 0.35 | 0.99 | 0.63 | 0.37 | 0.95 | 0.63 | 0.37 | 0.98 |
| rs2251002 | 7 | 30679369 | *CRHR2* | C | G | 0.65 | 0.35 | 0.99 | 0.63 | 0.37 | 0.95 | 0.63 | 0.37 | 0.98 |
| rs2284218 | 7 | 30680858 | *CRHR2* | T | C | 0.63 | 0.37 | 1.00 | 0.61 | 0.39 | 0.93 | 0.59 | 0.41 | 0.95 |
| rs2284219 | 7 | 30680961 | *CRHR2* | G | A | 0.63 | 0.37 | 1.00 | 0.61 | 0.39 | 0.96 | 0.60 | 0.40 | 0.98 |
| rs733453 | 7 | 30681298 | *CRHR2* | A | G | 0.63 | 0.37 | 1.00 | 0.61 | 0.39 | 0.93 | 0.59 | 0.41 | 0.95 |
| rs2267715 | 7 | 30682612 | *CRHR2* | A | G | 0.63 | 0.37 | 1.00 | 0.61 | 0.39 | 0.96 | 0.60 | 0.40 | 0.99 |
| rs12533248 | 7 | 30685114 | *CRHR2* | A | G | 0.65 | 0.35 | 0.97 | 0.63 | 0.37 | 0.95 | 0.62 | 0.38 | 0.97 |
| rs1076294 | 7 | 30686493 | *CRHR2* | C | G | 0.55 | 0.45 | 0.70 | 0.54 | 0.46 | 0.72 | 0.53 | 0.47 | 0.72 |
| rs255097 | 7 | 30693484 | *CRHR2* | A | G | 0.63 | 0.37 | 0.92 | 0.61 | 0.39 | 0.90 | 0.60 | 0.40 | 0.88 |
| rs255098 | 7 | 30693852 | *CRHR2* | A | G | 0.63 | 0.37 | 0.89 | 0.61 | 0.39 | 0.88 | 0.60 | 0.40 | 0.87 |
| rs255099 | 7 | 30694528 | *CRHR2* | A | G | 0.63 | 0.37 | 0.86 | 0.61 | 0.39 | 0.86 | 0.60 | 0.40 | 0.86 |
| rs255100 | 7 | 30695433 | *CRHR2* | T | A | 0.63 | 0.37 | 0.84 | 0.61 | 0.39 | 0.82 | 0.60 | 0.40 | 0.82 |
