# Supplementary material for: Genome-Wide Association Analysis in Asthma Subjects Identifies SPATS2L as a Novel Bronchodilator Response Gene
Source: PLoS Genet. 2012 Jul 5;8(7):e1002824. doi: 10.1371/journal.pgen.1002824 (PMC3390407; doi:10.1371/journal.pgen.1002824)
Supplement: Table S8 — Primary GWAS 1000GP Imputed SNP P-values and Beta coefficients for SNPs that have nominally significant p-values (<0.05), and are in, or within 50,000 KB of, genes (i.e. ADRB2, ADCY9, CRHR2, ARG1) previously identified as being associated with BDR. There were no such SNPs near ADRB2. CARE and ACRN P-values are 1-sided based on the direction in CAMP/LOCCS/LODO/Sepracor. SE = Standard Error for corresponding Beta coefficient. Combined P-values were obtained using the Liptak method, with weights proportional to population size. (DOCX) [file pgen.1002824.s015.docx]

|  |  | CAMP/LOCCS/LODO/Sepracor | | | CARE | | | ACRN | | |  |
| --- | --- | --- | --- | --- | --- | --- | --- | --- | --- | --- | --- |
| SNP | Reference Allele | BETA | SE | P | BETA | SE | P | BETA | SE | P | Combined P-value |
| rs2531993 | G | 2.72 | 1.03 | 8.5E-03 | -1.28 | 1.32 | 8.3E-01 | 0.19 | 1.89 | 4.6E-01 | 1.5E-02 |
| rs710893 | G | 2.55 | 1.03 | 1.4E-02 | -1.55 | 1.30 | 8.8E-01 | 0.15 | 1.87 | 4.7E-01 | 2.6E-02 |
| rs2230742 | G | 2.20 | 1.02 | 3.1E-02 | -1.41 | 1.28 | 8.6E-01 | 0.48 | 1.85 | 4.0E-01 | 4.8E-02 |
| rs2531992 | G | 2.57 | 1.03 | 1.3E-02 | -1.52 | 1.29 | 8.8E-01 | 0.19 | 1.84 | 4.6E-01 | 2.4E-02 |
| chr16:3962639 | T | 2.76 | 1.02 | 7.0E-03 | -1.55 | 1.24 | 8.9E-01 | 0.34 | 1.71 | 4.2E-01 | 1.4E-02 |
| rs9935335 | T | 2.24 | 0.95 | 1.8E-02 | -1.35 | 1.24 | 8.6E-01 | -0.03 | 1.67 | 5.1E-01 | 3.3E-02 |
| rs2531991 | A | 2.14 | 0.85 | 1.2E-02 | -1.06 | 1.17 | 8.2E-01 | 0.25 | 1.75 | 4.4E-01 | 2.0E-02 |
| rs2238436 | G | 2.14 | 0.85 | 1.2E-02 | -1.06 | 1.16 | 8.2E-01 | 0.26 | 1.75 | 4.4E-01 | 2.0E-02 |
| rs2072346 | T | 2.44 | 0.94 | 9.6E-03 | -1.24 | 1.21 | 8.5E-01 | 0.35 | 1.64 | 4.2E-01 | 1.6E-02 |
| rs2240735 | T | 2.14 | 0.85 | 1.2E-02 | -1.05 | 1.16 | 8.2E-01 | 0.26 | 1.73 | 4.4E-01 | 2.0E-02 |
| rs2601775 | G | 2.50 | 0.88 | 4.7E-03 | -1.16 | 1.14 | 8.5E-01 | 0.66 | 1.69 | 3.5E-01 | 7.9E-03 |
| rs2531989 | A | 2.98 | 1.00 | 2.9E-03 | -1.34 | 1.17 | 8.7E-01 | 0.72 | 1.60 | 3.3E-01 | 5.1E-03 |
| rs2531988 | G | 3.10 | 1.00 | 2.0E-03 | -1.23 | 1.19 | 8.5E-01 | 1.00 | 1.62 | 2.7E-01 | 3.2E-03 |
| rs12922685 | G | 2.99 | 1.00 | 2.7E-03 | -1.33 | 1.16 | 8.7E-01 | 0.72 | 1.59 | 3.3E-01 | 4.9E-03 |
| rs2531986 | C | 2.61 | 0.96 | 6.4E-03 | -1.24 | 1.18 | 8.5E-01 | 0.42 | 1.59 | 4.0E-01 | 1.1E-02 |
| rs2238439 | G | 2.52 | 0.88 | 4.2E-03 | -1.13 | 1.12 | 8.4E-01 | 0.66 | 1.63 | 3.4E-01 | 6.9E-03 |
| rs2041248 | G | 3.07 | 1.00 | 2.2E-03 | -1.28 | 1.16 | 8.6E-01 | 0.83 | 1.53 | 2.9E-01 | 3.7E-03 |
| rs2041247 | C | 3.05 | 1.00 | 2.3E-03 | -1.29 | 1.14 | 8.7E-01 | 0.71 | 1.52 | 3.2E-01 | 4.2E-03 |
| rs933392 | G | 3.09 | 1.00 | 2.2E-03 | -1.29 | 1.15 | 8.7E-01 | 0.71 | 1.52 | 3.2E-01 | 3.9E-03 |
| rs2531983 | C | 2.91 | 0.98 | 3.1E-03 | -1.27 | 1.19 | 8.6E-01 | 0.50 | 1.53 | 3.7E-01 | 5.6E-03 |
| rs12325363 | G | -2.00 | 1.07 | 6.1E-02 | 0.59 | 1.27 | 6.8E-01 | -1.67 | 1.32 | 1.0E-01 | 4.8E-02 |
| rs2159296 | G | -1.63 | 0.84 | 5.2E-02 | -1.05 | 0.96 | 1.4E-01 | -0.69 | 1.07 | 2.6E-01 | 3.0E-02 |
| rs2532022 | G | -1.65 | 0.84 | 4.9E-02 | -1.05 | 0.96 | 1.4E-01 | -0.69 | 1.07 | 2.6E-01 | 2.9E-02 |
| rs2532020 | G | -1.63 | 0.84 | 5.2E-02 | -1.05 | 0.96 | 1.4E-01 | -0.68 | 1.07 | 2.6E-01 | 3.0E-02 |
| rs2532019 | T | -1.62 | 0.84 | 5.3E-02 | -1.05 | 0.96 | 1.4E-01 | -0.66 | 1.06 | 2.7E-01 | 3.1E-02 |
| rs2601813 | C | -1.62 | 0.84 | 5.3E-02 | -1.05 | 0.96 | 1.4E-01 | -0.65 | 1.06 | 2.7E-01 | 3.1E-02 |
| rs6500578 | T | -1.62 | 0.84 | 5.3E-02 | -1.05 | 0.96 | 1.4E-01 | -0.65 | 1.06 | 2.7E-01 | 3.1E-02 |
| rs2781640 | G | 2.20 | 0.90 | 1.4E-02 | 0.35 | 1.18 | 3.8E-01 | -2.46 | 1.33 | 9.7E-01 | 3.6E-02 |
| rs2749929 | T | 2.27 | 0.91 | 1.2E-02 | 0.33 | 1.09 | 3.8E-01 | -1.95 | 1.22 | 9.4E-01 | 2.8E-02 |
| rs2781646 | A | 2.27 | 0.91 | 1.3E-02 | 0.45 | 1.09 | 3.4E-01 | -1.93 | 1.22 | 9.4E-01 | 2.7E-02 |
| rs2608909 | A | 2.21 | 0.90 | 1.4E-02 | 0.46 | 1.09 | 3.4E-01 | -1.93 | 1.22 | 9.4E-01 | 3.0E-02 |
| rs2608910 | T | 2.21 | 0.90 | 1.4E-02 | 0.48 | 1.09 | 3.3E-01 | -1.93 | 1.22 | 9.4E-01 | 2.9E-02 |
| rs2781647 | G | 2.20 | 0.90 | 1.4E-02 | 0.49 | 1.09 | 3.2E-01 | -1.94 | 1.22 | 9.4E-01 | 3.0E-02 |
| rs2608912 | T | 2.19 | 0.90 | 1.5E-02 | 0.72 | 1.09 | 2.5E-01 | -1.98 | 1.22 | 9.5E-01 | 2.9E-02 |
| rs2608913 | T | 2.19 | 0.90 | 1.5E-02 | 0.72 | 1.09 | 2.5E-01 | -1.98 | 1.22 | 9.5E-01 | 2.9E-02 |
| rs2608914 | T | 2.26 | 0.90 | 1.2E-02 | 0.78 | 1.08 | 2.4E-01 | -1.99 | 1.22 | 9.5E-01 | 2.3E-02 |
| rs2608915 | A | 2.19 | 0.90 | 1.5E-02 | 0.72 | 1.09 | 2.5E-01 | -1.98 | 1.22 | 9.5E-01 | 2.9E-02 |
| rs2608916 | T | 2.19 | 0.90 | 1.5E-02 | 0.72 | 1.09 | 2.5E-01 | -1.98 | 1.22 | 9.5E-01 | 2.9E-02 |
| rs2608917 | A | 2.19 | 0.90 | 1.5E-02 | 0.72 | 1.09 | 2.5E-01 | -1.98 | 1.22 | 9.5E-01 | 2.9E-02 |
| rs2608918 | C | 2.26 | 0.90 | 1.2E-02 | 0.78 | 1.08 | 2.4E-01 | -1.99 | 1.22 | 9.5E-01 | 2.3E-02 |
| rs2608919 | A | 2.20 | 0.90 | 1.5E-02 | 0.76 | 1.09 | 2.4E-01 | -1.84 | 1.20 | 9.4E-01 | 2.8E-02 |
| rs2608920 | T | 2.20 | 0.90 | 1.5E-02 | 0.76 | 1.09 | 2.4E-01 | -1.84 | 1.20 | 9.4E-01 | 2.8E-02 |
| rs2608921 | C | 2.17 | 0.90 | 1.6E-02 | 0.75 | 1.09 | 2.4E-01 | -1.82 | 1.20 | 9.3E-01 | 3.0E-02 |
| rs2446213 | T | 2.20 | 0.90 | 1.5E-02 | 0.76 | 1.09 | 2.4E-01 | -1.83 | 1.20 | 9.4E-01 | 2.8E-02 |
| rs2608983 | T | 2.20 | 0.90 | 1.5E-02 | 0.76 | 1.09 | 2.4E-01 | -1.83 | 1.20 | 9.4E-01 | 2.8E-02 |
| rs2749932 | C | 2.20 | 0.90 | 1.5E-02 | 0.76 | 1.09 | 2.4E-01 | -1.83 | 1.20 | 9.4E-01 | 2.8E-02 |
| rs2608982 | G | 2.44 | 1.09 | 2.5E-02 | 1.13 | 1.20 | 1.7E-01 | -1.82 | 1.36 | 9.1E-01 | 3.7E-02 |
| rs2608981 | T | 2.20 | 0.90 | 1.5E-02 | 0.76 | 1.09 | 2.4E-01 | -1.83 | 1.20 | 9.4E-01 | 2.8E-02 |
| rs28612296 | G | 2.27 | 0.90 | 1.2E-02 | 0.82 | 1.08 | 2.2E-01 | -1.84 | 1.20 | 9.4E-01 | 2.2E-02 |
| rs2781649 | A | 2.20 | 0.90 | 1.5E-02 | 0.76 | 1.09 | 2.4E-01 | -1.83 | 1.20 | 9.4E-01 | 2.8E-02 |
| rs2781650 | A | 2.20 | 0.90 | 1.5E-02 | 0.76 | 1.09 | 2.4E-01 | -1.83 | 1.20 | 9.4E-01 | 2.8E-02 |
| rs2781651 | A | 2.27 | 0.90 | 1.2E-02 | 0.82 | 1.08 | 2.2E-01 | -1.84 | 1.20 | 9.4E-01 | 2.2E-02 |
| rs2781652 | A | 2.20 | 0.90 | 1.5E-02 | 0.76 | 1.09 | 2.4E-01 | -1.82 | 1.20 | 9.4E-01 | 2.8E-02 |
| rs2749933 | C | 2.20 | 0.90 | 1.5E-02 | 0.77 | 1.09 | 2.4E-01 | -1.83 | 1.20 | 9.3E-01 | 2.8E-02 |
| rs2608977 | T | 2.20 | 0.90 | 1.5E-02 | 0.77 | 1.09 | 2.4E-01 | -1.83 | 1.20 | 9.3E-01 | 2.8E-02 |
| rs2608976 | T | 2.20 | 0.90 | 1.5E-02 | 0.77 | 1.09 | 2.4E-01 | -1.83 | 1.20 | 9.3E-01 | 2.8E-02 |
| rs2608975 | G | 2.20 | 0.90 | 1.5E-02 | 0.77 | 1.09 | 2.4E-01 | -1.83 | 1.21 | 9.3E-01 | 2.8E-02 |
| rs2781654 | C | 2.23 | 0.91 | 1.5E-02 | 0.80 | 1.23 | 2.6E-01 | -2.03 | 1.35 | 9.3E-01 | 2.7E-02 |
| rs9375818 | G | 2.57 | 1.08 | 1.8E-02 | 1.40 | 1.46 | 1.7E-01 | -2.33 | 1.67 | 9.2E-01 | 2.8E-02 |
| rs6929820 | T | 2.56 | 0.93 | 5.9E-03 | 0.87 | 1.31 | 2.5E-01 | -2.10 | 1.45 | 9.3E-01 | 1.2E-02 |
| rs2781656 | C | 2.30 | 0.91 | 1.2E-02 | 0.85 | 1.10 | 2.2E-01 | -1.85 | 1.26 | 9.3E-01 | 2.2E-02 |
| rs2749935 | A | 2.30 | 0.81 | 4.7E-03 | -0.45 | 0.95 | 6.8E-01 | -1.94 | 1.15 | 9.5E-01 | 1.8E-02 |
| rs2781659 | A | 2.17 | 0.80 | 7.0E-03 | -0.22 | 1.09 | 5.8E-01 | -1.41 | 1.22 | 8.8E-01 | 1.7E-02 |
| rs2781660 | T | 2.17 | 0.80 | 6.9E-03 | -0.22 | 1.09 | 5.8E-01 | -1.41 | 1.22 | 8.8E-01 | 1.7E-02 |
| rs2781661 | G | 2.17 | 0.80 | 6.9E-03 | -0.22 | 1.09 | 5.8E-01 | -1.41 | 1.22 | 8.8E-01 | 1.7E-02 |
| rs2781662 | A | 2.17 | 0.80 | 6.9E-03 | -0.22 | 1.09 | 5.8E-01 | -1.41 | 1.22 | 8.8E-01 | 1.7E-02 |
| rs2781663 | T | 2.17 | 0.80 | 6.9E-03 | -0.22 | 1.09 | 5.8E-01 | -1.40 | 1.22 | 8.7E-01 | 1.7E-02 |
| rs2608898 | C | 2.11 | 0.81 | 8.8E-03 | -0.24 | 1.09 | 5.9E-01 | -1.45 | 1.23 | 8.8E-01 | 2.1E-02 |
| rs2781664 | T | 2.31 | 0.88 | 8.6E-03 | -0.49 | 1.17 | 6.6E-01 | -1.55 | 1.33 | 8.8E-01 | 2.2E-02 |
| rs2781665 | A | 2.43 | 0.88 | 5.7E-03 | -0.38 | 1.19 | 6.2E-01 | -1.40 | 1.32 | 8.5E-01 | 1.4E-02 |
| rs2608897 | C | 2.13 | 0.78 | 6.3E-03 | -0.26 | 1.08 | 5.9E-01 | -1.37 | 1.22 | 8.7E-01 | 1.6E-02 |
| rs2781666 | G | 2.36 | 0.86 | 5.9E-03 | -0.50 | 1.16 | 6.7E-01 | -1.40 | 1.31 | 8.6E-01 | 1.6E-02 |
| rs2781667 | C | 2.16 | 0.78 | 5.9E-03 | -0.26 | 1.08 | 6.0E-01 | -1.30 | 1.22 | 8.6E-01 | 1.4E-02 |
| rs1076291 | G | 1.67 | 0.78 | 3.3E-02 | -0.70 | 0.89 | 7.8E-01 | 1.11 | 1.00 | 1.3E-01 | 3.1E-02 |
| rs1076292 | G | 1.67 | 0.78 | 3.3E-02 | -0.70 | 0.89 | 7.8E-01 | 1.11 | 1.00 | 1.3E-01 | 3.1E-02 |
| rs2251002 | C | 1.67 | 0.78 | 3.3E-02 | -0.70 | 0.89 | 7.8E-01 | 1.11 | 1.00 | 1.3E-01 | 3.1E-02 |
| rs2284218 | T | 1.53 | 0.77 | 4.7E-02 | -0.85 | 0.87 | 8.3E-01 | 1.35 | 1.01 | 9.1E-02 | 4.3E-02 |
| rs2284219 | G | 1.53 | 0.77 | 4.7E-02 | -0.82 | 0.86 | 8.3E-01 | 1.34 | 0.99 | 8.9E-02 | 4.3E-02 |
| rs733453 | A | 1.54 | 0.77 | 4.7E-02 | -0.85 | 0.87 | 8.3E-01 | 1.34 | 1.01 | 9.2E-02 | 4.3E-02 |
| rs2267715 | A | 1.54 | 0.77 | 4.7E-02 | -0.82 | 0.86 | 8.3E-01 | 1.33 | 0.99 | 9.0E-02 | 4.2E-02 |
| rs12533248 | A | 1.71 | 0.79 | 3.0E-02 | -0.89 | 0.87 | 8.5E-01 | 1.31 | 1.01 | 9.8E-02 | 2.9E-02 |
| rs1076294 | C | 1.96 | 0.89 | 2.8E-02 | -1.26 | 0.97 | 9.0E-01 | 1.49 | 1.14 | 9.6E-02 | 3.0E-02 |
| rs255097 | A | 1.60 | 0.80 | 4.7E-02 | -0.86 | 0.89 | 8.3E-01 | 1.35 | 1.05 | 1.0E-01 | 4.4E-02 |
| rs255098 | A | 1.61 | 0.82 | 4.8E-02 | -0.87 | 0.90 | 8.3E-01 | 1.36 | 1.06 | 1.0E-01 | 4.5E-02 |
| rs255099 | A | 1.63 | 0.83 | 5.0E-02 | -0.89 | 0.91 | 8.4E-01 | 1.37 | 1.07 | 1.0E-01 | 4.7E-02 |
| rs255100 | T | 1.65 | 0.84 | 5.1E-02 | -0.93 | 0.93 | 8.4E-01 | 1.39 | 1.10 | 1.0E-01 | 4.8E-02 |
