# Supplementary material for: Genome-Wide Association Analysis in Asthma Subjects Identifies SPATS2L as a Novel Bronchodilator Response Gene
Source: PLoS Genet. 2012 Jul 5;8(7):e1002824. doi: 10.1371/journal.pgen.1002824 (PMC3390407; doi:10.1371/journal.pgen.1002824)
Supplement: Text S1 — Detailed subject characteristics and genotype data quality control. (DOC) [file pgen.1002824.s016.doc]

**Detailed Subject Characteristics**

1. **Childhood Asthma Management Program (CAMP).** This population is composed of non-Hispanic white subjects from a clinical trial that followed 1,041 asthmatic children for 4-6 years (4.3 years on average) [1]. Stringent inclusion criteria ensured that participants had mild to moderate asthma, which was assessed as having asthma symptoms at least twice per week, using asthma medication daily, or using an inhaled bronchodilator twice per week for six or more months of the year prior to recruitment. CAMP subjects had increased airway responsiveness, as established by a 20% or greater decrease in FEV_1_ after administration of ≤12.5mg/dl of methacholine. Spirometry was performed according to American Thoracic Society recommendations with a volume-displacement spirometer [1] and was performed by pulmonary function technicians trained and certified specifically for the CAMP protocol and procedures at least 4 hours after the use of a short-acting bronchodilator and 24 hours after the use of a long-acting bronchodilator. After administration of a bronchodilator (two puffs albuterol by metered-dose inhaler), the minimal elapsed time before the postbronchodilator test was 15 minutes. BDR was defined as the percentage change in FEV_1_ after bronchodilator administration. Baseline BDR was measured after a four-week wash-out period at the randomization time-point. CAMP participants and their parents provided DNA for genetic studies. Informed consent was obtained from all CAMP participants and their parents. The studies were approved by the Institutional Review Board of Partners’ Healthcare and by all eight CAMP clinical centers and the CAMP Data Coordinating Center.
2. **Leukotriene Modifier or Corticosteroid Salmeterol (LOCCS).** This study was conducted by the American Lung Association Asthma Clinical Research Centers [2]. All subjects (n = 159) completed a 4-6 week run-in on inhaled fluticasone propionate (corticosteroid) after which they had well-controlled asthma as determined by a Juniper Asthma Control Questionnaire score of < 1.5. All participants had prebronchodilator FEV_1_ of ≥ 80% of predicted value, and ≥ 12% bronchodilator reversibility or methacholine challenge (PC20) of ≤ 8mg/ml. Baseline BDR was measured after the run-in on fluticasone (100μg twice daily) using two puffs of Albuterol.
3. **Effectiveness of Low Dose Theophylline as an Add-on Treatment in Asthma (LODO).** This trial was conducted by the American Lung Association Asthma Clinical Research Centers [3]. Subjects had prebronchodilator FEV_1_ of ≥ 50% of predicted value [4], poorly controlled asthma (regardless of baseline treatment regimen) as measured by a score of ≥ 1.5 on the Juniper asthma control questionnaire [5,6]. Participants did not have a history of smoking, or other known illnesses and were on stable asthma medications prior to randomization. After a 1-2-week run-in on placebo, baseline BDR was measured using two puffs of Albuterol.
4. **Sepracor.** Subjects were adults recruited for a medication trial conducted by Sepracor, Inc., US [7,8] and diagnosed with moderate to severe asthma according to the American Thoracic Society criteria [9] with no significant comorbid medical conditions. Participants had prebronchodilator forced expiratory volume in one second (FEV_1_) of 40-85% of predicted normal values after at least eight hours without use of an inhaled short-acting beta-agonist (SABA). Subjects experienced a minimum of 15% reversibility of airway flow obstruction in response to the β-agonist or were challenged with a methacholine sensitivity test to confirm diagnosis. No oral or inhaled corticosteroids were administered during the six weeks leading up to the trial where baseline BDR was assessed.
5. **Asthma Clinical Research Network (ACRN).** The ACRN was established in 1993 by the National Heart, Lung and Blood Institute (NHLBI) to conduct multiple well designed clinical trials for rapid evaluation of new and existing therapeutic approaches to asthma and to disseminate laboratory and clinical findings to the health care community (http://www.acrn.org) [10,11]. Adults patients from the following ACRN trials were used for the current study: Beta Agonist in Mild Asthma Study (BAGS; n=98) [12], Dose of Inhaled Corticosteroids with Equisystemic Effects (DICE; n=7) [13], Improving Asthma Control Trial (IMPACT; n=106) [14], and Predicting Responses for Inhaled Corticosteroid Efficacy (PRICE; n=30) [15]. BAGS subjects were selected to have mild asthma, and after a six-week run-in on placebo, baseline BDR was measured using two puffs of Albuterol. DICE subjects were selected to have mild-to-moderate asthma, and baseline BDR was measured at the time of recruitment using two puffs of Albuterol. IMPACT subjects were selected to have mild persistent asthma, and after a four-week run-in on placebo, baseline BDR was measured using four puffs of Albuterol. PRICE subjects were selected to have mild persistent asthma, and after a two-week run-in on placebo, baseline BDR was measured using four puffs of Albuterol.
6. **Childhood Asthma Research and Education (CARE).** The CARE Network was established in 1999 by the NHLBI to evaluate treatments for children with asthma (http://www.asthma-carenet.org) [16-18]. Subjects from the following CARE trials were used for the current study: Characterizing the Response to a Leukotriene Receptor Antagonist and an Inhaled Corticosteroid (CLIC; n=71) [18], Pediatric Asthma Controller Trial (PACT; n=116) [19], and Montelukast or Azithromycin for Reduction of Inhaled Corticosteroids in Childhood Asthma (MARS; n=20) [20]. CLIC subjects were children selected to have mild to moderate persistent asthma, and baseline BDR was measured at the time of recruitment **off controller medications for at least 2-weeks** using four puffs of Albuterol. PACT subjects were children selected to have mild to moderate persistent asthma, and after a 2-to-4-week run-in on placebo, baseline BDR was measured using four puffs of Albuterol. MARS subjects were children selected to have moderate to severe persistent asthma, and were **on controller inhaled corticosteroids** when baseline BDR was measured using four puffs of Albuterol.

**Detailed Genotype Data Quality Control**

1. **CAMP.** Genome-wide SNP genotyping for 422 Caucasian CAMP subjects and their families was performed on Illumina’s HumanHap550v3 Genotyping BeadChip (Illumina, Inc., San Diego, CA). Samples and markers passed stringent quality control (QC) standards that have been described previously [21]. An additional 211 samples were genotyped on the Illumina Infinium HD Human610-Quad BeadChip at the Channing Laboratory and merged with those genotyped on the HumanHap550v3 platform. Of 1348 unique CAMP subjects that were successfully genotyped on either platform, 4 had a sample run on both the HumanHap550 and the Human610-Quad and were used for QC in merging the data. Passing subjects all had >96.5% completion rate, while the average completion rate was 99.8%. Concordance among 4 passing replicate samples within the 610 dataset is 99.99%; concordance among 14 replicates of 5 subjects within the 550 dataset was >99.8% for any pair. Concordance among replicates between the 610 and 550 was an average of 99.99% for all pairwise comparisons, with a minimum concordance of 99.89%. Each subject’s genotypic gender, as determined by PLINK’s sexcheck algorithm, agrees with the phenotypic gender. Identity of each sample was checked by comparing up to 1180 markers that overlap between the BeadChips and previous genotyping performed at the Channing Laboratory. Each sample had 97.8 to 100% concordance, confirming sample identity. The average Mendelian error rate in nuclear families, determined using PLINK, was 0.008% in the passing subjects. Of 592,532 SNP markers present on the 610 chip, 9022 were failed for one or more of the following reasons: 1) probe sequences did not map uniquely to the hg18 genome build, 2) poor cluster separation as manually reviewed in Illumina BeadStudio software, 3) –log10(pval) for Hardy-Weinberg equilibrium >= 8, or 4) genotype completion rate < 95%. Of 561,466 SNPs present on the 550 chip, 16,419 were failed these reasons, or for 5) Mendelian error count >=5, or 6) minor allele frequency (MAF) = 0. Only markers that passed QC on both chips were included in the final dataset. Finally, 1819 markers were excluded based on MAF discordance among the replicates run on both chips. The final number of passing markers in the merged dataset was 528,890.
2. **LOCCS/LODO/Sepracor.** Of 1066 bronchodilator study samples run on the Illumina Human610-Quad genotyping BeadChip, 1009 unique subjects passed.  Passing subjects all had greater than 97% completion rate, while the average completion rate was 99.9%. Each subject’s genotypic gender, as determined by PLINK’s sexcheck algorithm, agrees with the phenotypic gender. rgGRR [22] was used to evaluate allele sharing between each subject pair, and subjects found to be related were flagged in the dataset and excluded further from the analysis. The identity of each sample was confirmed by comparing up to 2052 markers that overlap between the 610 chip and previous genotyping performed at the Channing Laboratory. In addition to the bronchodilator subjects, 5 QC subjects were run with replicate samples. Observed average concordance among 53 replicate samples (9 to 12 replicates per subject for 5 subjects) within the 610 dataset was 99.99%. Of 592,532 SNP markers present on the chip, 10,241 were failed for one or more of the following reasons: 1) probe sequences did not map uniquely to the hg18 genome build, 2) p-value for Hardy-Weinberg equilibrium <= 10^-6, 3) completion rate < 95%, 4) >1% discordance observed among sample replicates, 5) >2% discordance with previous genotyping on other platforms, and 6) minor allele frequency of zero. The final number of passing markers in the dataset was 582,291 or 98.3% of the available markers. Intensity-only probes on the chip were excluded.
3. **CARE/ACRN.** DNA samples from 1,604 CARE probands and their parents, and 1059 ACRN subjects were genotyped on the Affymetrix 6.0 genotyping chip by Affymetrix, Inc. (Santa Clara, CA). Of these white subjects, 721 from ACRN and 922 from CARE had a genotyping completion rate >95%. Of 934,940 SNP markers present on the 6.0 chip, 152,960 in ACRN and 186,890 in CARE were failed based on not mapping uniquely to the hg18 genome build, having >12 discordances among sample replicate pairs, having >3 Mendelian error counts in trios (applicable to CARE only), having genotype missing rate >5%, or having HWE p-value <1E-06. Consistency between genotype and stated gender was verified in PLINK. Lack of cryptic subject relatedness was verified using rgGRR [22].

# References

1. Childhood Asthma Management Program Research Group. (1999) The Childhood Asthma Management Program (CAMP): design, rationale, and methods. Control Clin Trials 20: 91-120.

2. Peters SP, Anthonisen N, Castro M, Holbrook JT, Irvin CG, et al. (2007) Randomized comparison of strategies for reducing treatment in mild persistent asthma. N Engl J Med 356: 2027-2039.

3. American Lung Association Asthma Clinical Research Centers. (2007) Clinical trial of low-dose theophylline and montelukast in patients with poorly controlled asthma. Am J Respir Crit Care Med 175: 235-242.

4. Hankinson JL, Odencrantz JR, Fedan KB (1999) Spirometric reference values from a sample of the general U.S. population. Am J Respir Crit Care Med 159: 179-187.

5. Juniper EF, Bousquet J, Abetz L, Bateman ED (2006) Identifying 'well-controlled' and 'not well-controlled' asthma using the Asthma Control Questionnaire. Respir Med 100: 616-621.

6. Juniper EF, O'Byrne PM, Guyatt GH, Ferrie PJ, King DR (1999) Development and validation of a questionnaire to measure asthma control. Eur Respir J 14: 902-907.

7. Silverman ES, Palmer LJ, Subramaniam V, Hallock A, Mathew S, et al. (2004) Transforming growth factor-beta1 promoter polymorphism C-509T is associated with asthma. Am J Respir Crit Care Med 169: 214-219.

8. Silverman ES, Baron RM, Palmer LJ, Le L, Hallock A, et al. (2002) Constitutive and cytokine-induced expression of the ETS transcription factor ESE-3 in the lung. Am J Respir Cell Mol Biol 27: 697-704.

9. American Thoracic Society. (1995) Standards for the diagnosis and care of patients with chronic obstructive pulmonary disease. Am J Respir Crit Care Med 152: S77-121.

10. Sutherland ER, Lehman EB, Teodorescu M, Wechsler ME (2009) Body mass index and phenotype in subjects with mild-to-moderate persistent asthma. J Allergy Clin Immunol 123: 1328-1334 e1321.

11. Szefler SJ, Martin RJ, King TS, Boushey HA, Cherniack RM, et al. (2002) Significant variability in response to inhaled corticosteroids for persistent asthma. J Allergy Clin Immunol 109: 410-418.

12. Drazen JM, Israel E, Boushey HA, Chinchilli VM, Fahy JV, et al. (1996) Comparison of regularly scheduled with as-needed use of albuterol in mild asthma. Asthma Clinical Research Network. N Engl J Med 335: 841-847.

13. Martin RJ, Szefler SJ, Chinchilli VM, Kraft M, Dolovich M, et al. (2002) Systemic effect comparisons of six inhaled corticosteroid preparations. Am J Respir Crit Care Med 165: 1377-1383.

14. Boushey HA, Sorkness CA, King TS, Sullivan SD, Fahy JV, et al. (2005) Daily versus as-needed corticosteroids for mild persistent asthma. N Engl J Med 352: 1519-1528.

15. Martin RJ, Szefler SJ, King TS, Kraft M, Boushey HA, et al. (2007) The Predicting Response to Inhaled Corticosteroid Efficacy (PRICE) trial. J Allergy Clin Immunol 119: 73-80.

16. Lemanske RF, Jr., Allen DB (1997) Choosing a long-term controller medication in childhood asthma. The proverbial two-edged sword. Am J Respir Crit Care Med 156: 685-687.

17. Lemanske RF, Jr., Mauger DT, Sorkness CA, Jackson DJ, Boehmer SJ, et al. (2010) Step-up therapy for children with uncontrolled asthma receiving inhaled corticosteroids. N Engl J Med 362: 975-985.

18. Szefler SJ, Phillips BR, Martinez FD, Chinchilli VM, Lemanske RF, et al. (2005) Characterization of within-subject responses to fluticasone and montelukast in childhood asthma. J Allergy Clin Immunol 115: 233-242.

19. Sorkness CA, Lemanske RF, Jr., Mauger DT, Boehmer SJ, Chinchilli VM, et al. (2007) Long-term comparison of 3 controller regimens for mild-moderate persistent childhood asthma: the Pediatric Asthma Controller Trial. J Allergy Clin Immunol 119: 64-72.

20. Strunk RC, Bacharier LB, Phillips BR, Szefler SJ, Zeiger RS, et al. (2008) Azithromycin or montelukast as inhaled corticosteroid-sparing agents in moderate-to-severe childhood asthma study. J Allergy Clin Immunol 122: 1138-1144 e1134.

21. Himes BE, Hunninghake GM, Baurley JW, Rafaels NM, Sleiman P, et al. (2009) Genome-wide association analysis identifies PDE4D as an asthma-susceptibility gene. Am J Hum Genet 84: 581-593.

22. Abecasis GR, Cherny SS, Cookson WO, Cardon LR (2001) GRR: graphical representation of relationship errors. Bioinformatics 17: 742-743.
